# Diel transcriptional response of a California Current plankton microbiome to light, low iron, and enduring viral infection

**DOI:** 10.1038/s41396-019-0472-2

**Published:** 2019-07-18

**Authors:** B. C. Kolody, J. P. McCrow, L. Zeigler Allen, F. O. Aylward, K. M. Fontanez, A. Moustafa, M. Moniruzzaman, F. P. Chavez, C. A. Scholin, E. E. Allen, A. Z. Worden, E. F. Delong, A. E. Allen

**Affiliations:** 1 Scripps Institution of Oceanography, University of California, San Diego, CA 92093 USA; 2 Microbial and Environmental Genomics Group, J. Craig Venter Institute, La Jolla, CA 92037 USA; 3 Department of Biological Sciences, Virginia Tech, Blacksburg, VA 24061 USA; 4 Department of Civil and Environmental Engineering, Massachusetts Institute of Technology, Cambridge, MA 02139 USA; 5 Department of Biology and Biotechnology Graduate Program, American University in Cairo, New Cairo, Egypt; 6 Monterey Bay Aquarium Research Institute, Moss Landing, CA 95039 USA; 7 Ocean EcoSystems Biology Unit, GEOMAR Helmholtz Centre for Ocean Research, Kiel, DE Germany; 8 Daniel K. Inouye Center for Microbial Oceanography: Research and Education (C-MORE), University of Hawaii, Honolulu, HI 96822 USA

**Keywords:** Water microbiology, Biogeochemistry, Microbial biooceanography

## Abstract

Phytoplankton and associated microbial communities provide organic carbon to oceanic food webs and drive ecosystem dynamics. However, capturing those dynamics is challenging. Here, an in situ, semi-Lagrangian, robotic sampler profiled pelagic microbes at 4 h intervals over ~2.6 days in North Pacific high-nutrient, low-chlorophyll waters. We report on the community structure and transcriptional dynamics of microbes in an operationally large size class (>5 μm) predominantly populated by dinoflagellates, ciliates, haptophytes, pelagophytes, diatoms, cyanobacteria (chiefly *Synechococcus)*, prasinophytes (chiefly *Ostreococcus)*, fungi, archaea, and proteobacteria. Apart from fungi and archaea, all groups exhibited 24-h periodicity in some transcripts, but larger portions of the transcriptome oscillated in phototrophs. Periodic photosynthesis-related transcripts exhibited a temporal cascade across the morning hours, conserved across diverse phototrophic lineages. Pronounced silica:nitrate drawdown, a high flavodoxin to ferredoxin transcript ratio, and elevated expression of other Fe-stress markers indicated Fe-limitation. Fe-stress markers peaked during a photoperiodically adaptive time window that could modulate phytoplankton response to seasonal Fe-limitation. Remarkably, we observed viruses that infect the majority of abundant taxa, often with total transcriptional activity synchronized with putative hosts. Taken together, these data reveal a microbial plankton community that is shaped by recycled production and tightly controlled by Fe-limitation and viral activity.

## Introduction

Phytoplankton productivity is essential for supporting marine food webs and represents a key variable in biogeochemical cycling and climate models [1]. Primary productivity is often determined locally by eddy-scale upwelling and molecular-scale interactions between bacteria, viruses, grazers, and phytoplankton [2, 3]. As a result of observational limitations of these molecular-scale processes in situ, much of our knowledge of phytoplankton physiology derives from laboratory-based experiments. Such studies have been instrumental in elucidating the basic biology and genetic potential of many individual phytoplankton [4–8], however, they cannot capture ecological interactions with uncultured community members or the influence of the advection dynamics found in nature.

Recently, the Environmental Sample Processor (ESP) [9], a robotic sampling device, has been deployed in a drifter configuration to follow sympatric populations of small (<5 µm) microbes within a particular water mass. These studies elucidated diel transcriptional rhythms first in the picophytoplankton, *Synechococcus* and *Ostreococcus* [10], and later in heterotrophic bacterioplankton [11] and viruses [12]. A comparison of drifts from diverse environments revealed a daily “cascade” of transcriptional activity across taxa that was conserved on ocean basin scales [13].

However, it is unknown whether these findings extend to the majority of eukaryotic primary producers (diatoms, haptophytes, pelagophytes, chlorophytes, etc.), planktonic predators such as ciliates and dinoflagellates, and particle-associated microbes. If diel transcriptional rhythms exist in these taxa, do similar functions co-occur across diverse lineages, or do they fall in a temporal cascade? In addition, it is unknown to what extent abiotic factors such as nutrient limitation affect the prevalence of diel transcription.

Here, we analyzed the large size-class filters associated with the initial deployment of the ESP [10], which collected whole-community RNA samples every 4 h over ~2.6 days in the central California Current upwelling system (cCCS). Because of the semi-Lagrangian nature of this ESP deployment (Fig. S1a), a coherent microbial community was observed in both size classes, providing a unique record of its diel response to changes in sunlight as well as in situ nutrient conditions and prevailing ecosystem dynamics. We asked whether the timing and function of diel transcription was conserved across diverse, uncultured protist lineages, as well as how productivity was affected by local iron stress. In addition, access to both filters allowed us to juxtapose free-living microbes with those putatively associated with particles in the same environment, and compare the role of viruses in both fractions.

## Materials and methods

Full methods, including methods for rRNA amplicon processing and phylogenetics, are described in Supplementary File 1, and raw data can be accessed at NCBI (BioProject accession number PRJNA492502; BioSample accession numbers SAMN10104964-SAMN10105011). Processed data can be found in Supplementary Datasets 1–12, and are described in Supplementary File 3. Briefly, 1 L samples were collected every ~4 h (16 samples in total) by an ESP suspended 23 m below a semi-Lagrangian surface float as previously described [10] from September 16–19, 2010. Seawater was size fractionated in situ onto large fraction (5 µm) and small fraction (0.22 µm) filters. cDNA for metatranscriptomes was prepared as described in Ottesen et al. [10] from ribosomal RNA-depleted total RNA [10].

Metatranscriptome quality control, trimming, filtration, and rRNA removal was conducted on large fraction Illumina reads and the previously reported small fraction 454 reads via the RNAseq Annotation Pipeline v0.4 (Fig. S2) [14]. Ab initio open-reading frames (ORFs) were predicted on assembled large fraction contigs and unassembled small fraction 454 reads. ORFs were annotated via BLASTP [15] alignment to a comprehensive protein database, *phyloDB* (Supplementary File 1). To avoid biases introduced by classifying ORFs based on best BLAST to potentially contaminated sequences, particularly important when using microbial reference transcriptomes obtained from non-axenic laboratory cultures, a Lineage Probability Index (LPI) was used to assign taxonomy [14, 16, 17]. LPI was calculated here as a value between 0 and 1 indicating lineage commonality among the top 95-percentile of sequences based on BLAST bit-score [14, 16]. For each taxa group, the references with the highest mean percent identity to ab initio ORFs and that recruited the most ORFs were used for nucleotide-space mapping. References with at least 1000 genes with at least five reads mapped were then considered for downstream analysis. Coverage statistics are provided in Fig. S3. Reference ORFs were hierarchically clustered together with ab initio ORFs from both fractions to form peptide ortholog groups and assigned a consensus annotation. ORFs with significantly periodic diel expression were identified using harmonic regression analysis (HRA) as previously described [10, 11, 13]. The Weighted Gene Correlation Network Analysis (WGCNA) R package [18] was used as previously described [13] to identify modules of conserved expression among ORFs and functional clusters.

## Results and discussion

The ESP was deployed offshore of Big Sur in the cCCS “transition zone” between nutrient-dense coastally-upwelled water and the oligotrophic open ocean. Despite sustaining highly productive fisheries, this region is characterized by frequent, variable levels of iron stress (Fe <0.2 nmol/kg; Fig. S4) and concordant high residual nitrate (5–15 µM) and low chlorophyll (1–2 µg/l; refs. [19–22]). We measured high nitrate (5-13 µM) and low-chlorophyll concentrations (<1 µM; Fig. S1b) in addition to a silica:nitrate ratio indicative of iron stress. Silica:nitrate ratios in the range of 0.8–1.1 are associated with iron limitation [20] and are thought to result from silica drawdown by iron-stressed diatoms [23]. In our study, this ratio was initially around 1 and dropped by an order of magnitude along the drift track (Fig. S1b). Molecular evidence, including expression of several low-iron response genes and a strikingly high flavodoxin:ferredoxin ratio, also supported iron limitation (Supplementary File 1, Fig. S5).

### Taxonomic structure of the active community

**Fig. 1:**
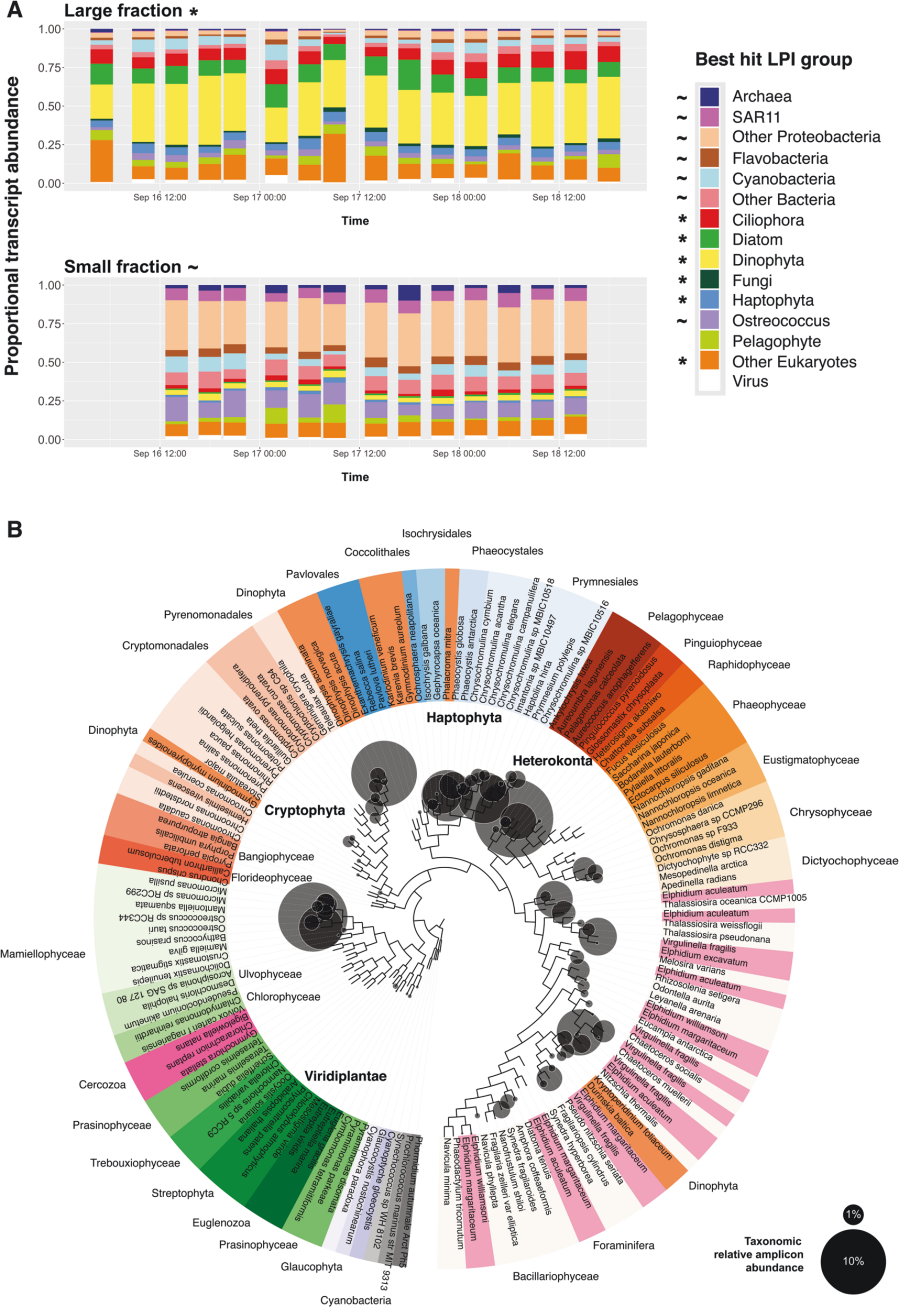
Major taxa found in the eastern North Pacific drift track. **a** Taxonomically annotated total community expression over time across size classes. Expression includes only non-organellar ORFs and is normalized by library (time point) within each fraction. Taxa grouping of each ab initio ORF is determined by best LPI hit. Asterisks and tildes denote taxa groups significantly enriched in the large and small size classes, respectively (edgeR, FDR <0.05). **b** Phylogenetic reconstruction of 16S rRNA gene reference sequences and distributions of active plastids represented in terms of cDNA-based amplicon relative abundances summed across all time points (circles; Supplementary Dataset 5). Circles representing relative amplicon abundance are superimposed over a reference phylogeny which is colored by taxonomy. Proximity of circles to the tips of the branches represents closeness of observed sequences to the references and circle sizes are proportional to normalized read abundance. Note that dinoflagellates known to have tertiary plastids are placed in this method according to the plastid origins (e.g., *Gymnodinium myriopyrenoides* and several *Dinophysis* species clading with cryptophytes [93])

The mRNA taxonomic assignments depicted an active community that was stable over time for both size fractions. This was the case at both coarse taxonomic (Fig. 1a) and genus (Fig. S6) levels, indicating that the drift track sampled a sympatric community of plankton. Large fraction mRNA activity was dominated by photosynthetic eukaryotes for which mapping to reference transcriptomes generally averaged <80% nucleotide identity (Figs. S7 and S8). In all, 45.9% of ab initio ORFs did not have any database match, a testament to the breadth of novel plankton diversity that remains uncultured, even in coastal regimes.

Dinoflagellates were the largest identifiable contributor to large size-class activity (34.8% of library normalized reads; Fig. 1a), with *Alexandrium*, *Karenia*, and *Karlodinium* each accounting for ∼20% of dinoflagellate mRNA (Fig. S6e). Phylogenetic analysis of 18S rRNA amplicons (Fig. S9) also depicted a community dominated by dinoflagellates, and 16S rRNA amplicons from chloroplasts (Fig. 1b) confirmed that many were photosynthetic. While copy number variation is a potential source of bias for 18S amplicon data [24], here our 18S community structure is largely in agreement with our transcript-based annotations. Non-plastid 16S rRNA amplicons from the large size class were dominated by cyanobacteria, *Bacteriodetes*, and Proteobacteria (Fig. S10).

Other major large fraction taxa included centric diatoms (10.3% of mRNA), ciliates (9.0%), metazoans (6.2%), haptophytes (5.2%), green algae (5.0%, primarily prasinophytes), *Synechococcus* (4.6%), and pelagophytes (4.5%). Diatoms and pelagophytes were overwhelmingly dominated by *Chaetoceros* (Fig. S6d) and *Pelagomonas* (Fig. S6g), respectively, while *Ostreococcus* and *Phaeocystis* dominated green algae and haptophytes to a lesser extent (Fig. S6a, f). Several of these taxa were previously shown to have high cell abundances in non-fractionated samples using quantitative methods during this drift and in other regional studies [25–28].

In order to address the entire community, we also used the size-fractionated data to distinguish putatively particle-associated (large fraction) from free-living (small fraction) taxa [29–31]. Fungi were significantly enriched in the large fraction (EdgeR FDR <0.05; log_2_FC = −5.2) and highly expressed a cellulose degrading glycoside hydrolase family 7 enzyme (Fig. 1a, Supplementary Data 6). Recently, fungi were shown to be among the most important eukaryotes on bathypelagic marine snow [32]. Hence, our results illustrate that their importance in particle ecology likely extends into the surface ocean. Likewise, many prokaryotes significantly enriched in the large fraction (EdgeR FDR < 0.05) have been commonly associated with particles, such as the *Cytophaga* (2.3% of bacterial expression) and *Planctomyces* (0.5%) (Supplementary Dataset 10). These taxa are important recyclers of structural and storage forms of carbon [33, 34], and we found active expression of bacterial organic matter degrading enzymes, such as glycoside hydrolase family 16 and secreted glycosyl hydrolases (Supplementary Dataset 6). Furthermore, the *comEA* and *comEC* gene clusters for the bacterial process of taking up exogenous DNA (competence) were enriched in the large fraction (EdgeR FDR < 0.05; Supplementary Dataset 8) and expressed across 11 large fraction bacterial genera. Particle-attached bacteria may have taken up exogenous DNA, potentially as a nutrient source, for DNA repair, or to increase genetic diversity [35], as seen in *Vibrios* attached to chitin [36].

### Patterns of community activity

In the large size class, total gene expression was often highly synchronized between members of a given taxonomic group (Fig. S11a), especially among prokaryotes. For example, flavobacteria and euryarchaeota ORFs showed strong ingroup correlation (Fig. S11a; Pearson’s *r* = 0.94, 0.93, respectively) and were most highly expressed at night (Fig. S11c, d). In total, HRA detected ten large-fraction taxa, including the diatom, *Skeletonema* sp., and the bacteria, *Roseobacter* sp., with total gene expression that followed smooth day/night oscillation with a period of 24 h (Fig. S12).

### Functional characterization of the active community

**Fig. 2:**
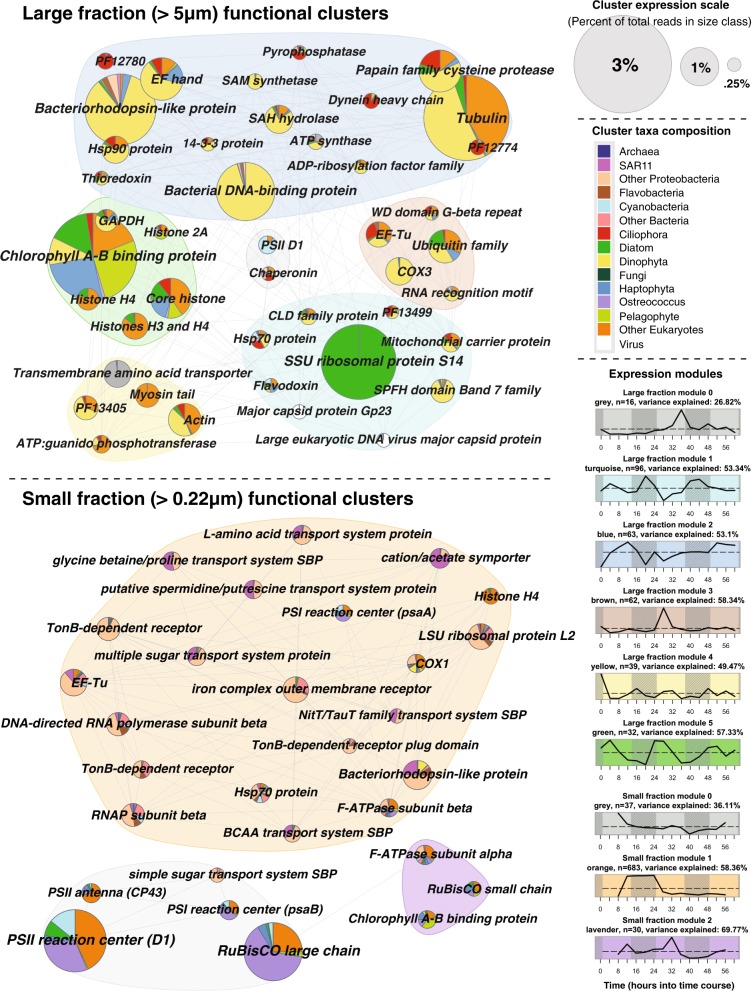
A comparison of functional diversity across the large and small size classes. Pies represent highly abundant (>0.25% total size-class expression) annotated functional clusters of ab initio ORFs. Pies are colored by relative taxonomic contribution and grouped by modules of similar expression as given by WGCNA

WGCNA on functional clusters established six unique patterns (“modules”) of gene expression over time in the large fraction and three in the small fraction (Fig. 2). WGCNA on nucleotide sequences aligned to reference transcriptomes recapitulated major functions but did not capture the same breadth of novel phylogenetic diversity that resulted from aligning amino acid sequences of ab initio ORFs (Fig. S13). Ab initio analysis established 344,615 unique ORFs which recruited ~107 million reads, whereas mapping reads to reference transcriptomes captured only ~15 million reads mapping to 168,349 reference ORFs.

In the large size class, the most obvious drivers of gene expression were taxonomy and day/night cycles. The most abundant cluster in large fraction module 1 (Fig. 2; turquoise; up at night) was a small subunit ribosomal protein, almost entirely composed of centric diatoms. Interestingly, prokaryotic and eukaryotic viral capsids both clustered into this night-up module. Large fraction module 2 (blue; erratic) echoed the shape of overall dinoflagellate activity (Fig. S11a), and major cluster annotations (e.g., tubulin, bacteriorhodopsin-like protein, and bacterial DNA-binding protein (the dinoflagellate equivalent of histones [37])) were dominated by dinoflagellates. Module 5 (green; peaks in the early morning), on the other hand, was dominated by photosynthesis-related annotations such as chlorophyll A–B binding protein and G3P dehydrogenase but also contained metazoan histones.

In the small fraction, the majority of cluster annotations were in module 1 (Fig. 2; orange; up during first night). Most functions related to growth (ribosomal proteins, RNA polymerase, and elongation factor Tu) and nutrient acquisition (branched-chain amino acid transporters, Nit Tau family transport system, and multiple sugar transport system) and were dominated by Proteobacteria. Photosynthesis-related transcription in the small fraction could mostly be attributed to *Ostreococcus*, “other eukaryotes”, and *Synechococcus*.

### Physiological response of phytoplankton to day/night cycles

In addition to identifying data-driven patterns of gene expression with WGCNA, we also sought to probe diel physiology by fitting gene expression to a sinusoid with a 24 h period (HRA). Large portions of photoautotroph transcriptomes have been observed to oscillate with a 24-h period, often by known circadian mechanisms (e.g., *Arabidopsis thaliana* [38], *Synechococcus elongatus* [39], and *Ostreococcus tauri* [40]). Most previous observations of this light response were performed in artificially stable laboratory settings (e.g., 12:12-h light:dark cycles), but Ottesen et al. [10] observed a high number of *Synechococcus* and *Ostreococcus* transcripts in the small size fraction of this drift, including key clock, respiration, and photoautotrophy genes, oscillating with a 24-h period in natural environments [10].

**Fig. 3:**
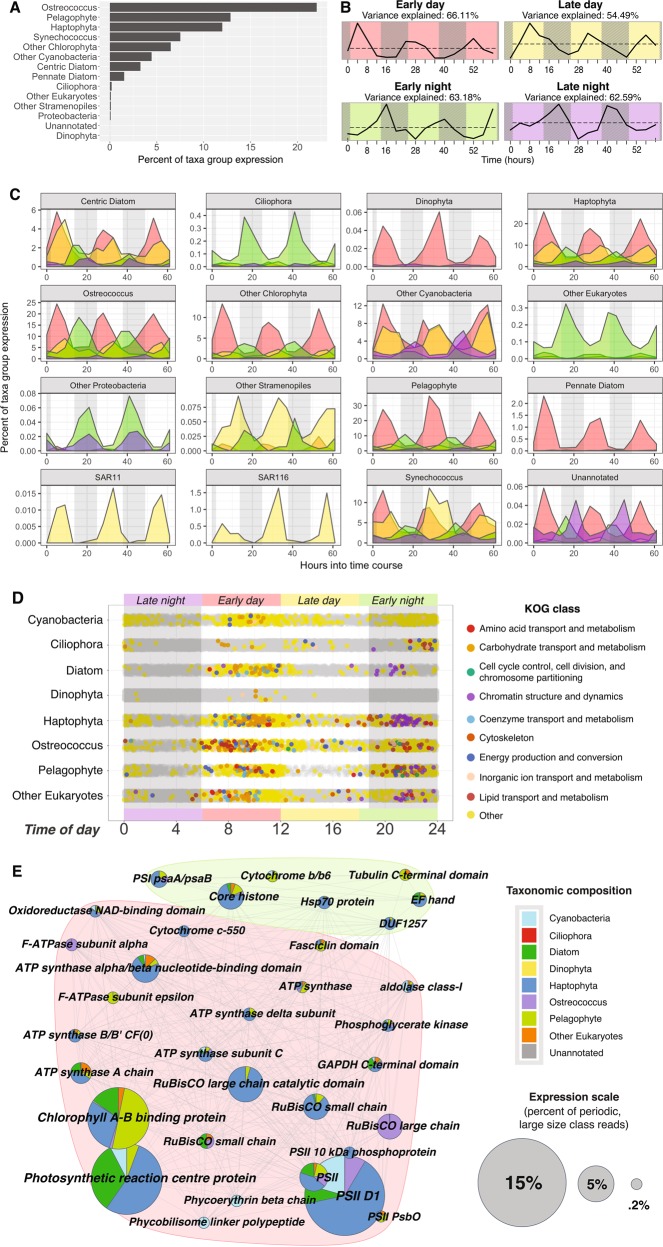
Timing, abundance, and diversity of significantly diel large fraction ab initio ORFs (HRA on taxa group normalized ORFs; FDR ≤0.1). ORFs are categorized into four, 6-h long bins based on peak expression time: early day (red, 6 a.m.–12 p.m.), late day (yellow, 12 p.m.–6 p.m.), early night (green, 6 p.m.–12 a.m.), and late night (purple, 12 a.m.–6 a.m.). **a** Percent of total expression found to be significantly periodic across taxa groups. **b** Data-driven WGCNA modules of significantly periodic large fraction ORFs independently recreate peak expression time bins. Subtitles show percent of variance that can explained by each module’s average expression profile. **c** Periodic expression colored by time of day bin across taxa groups. **d** Peak expression time of nuclear ORFs belonging to major phytoplankton players. Significantly periodic ORFs are depicted by colored dots, where colors correspond to KOG class; all other ORFs depicted in gray. The majority of periodic ORFs peak in early day (red; 54.8%) and early night (green; 31.2%). **e** Top annotated cluster annotations of significantly periodic large fraction ORFs. Pies are colored by relative taxonomic contribution (legend, right) and grouped by modules of similar expression (**b**)

Here, we expand this analysis to natural populations of large microbial eukaryotes for the first time. We observe significant diel transcriptional periodicity (FDR ≤ 0.1) in all active phytoplankton lineages, as well as in ciliates and some bacteria, and *Synechococcus* and *Ostreococcus* ORFs present in the large fraction (Fig. 3).

In addition, we detected phylogenetically novel Light-Oxygen-Voltage (LOV) domains, which have been implicated in transcriptional light response, including as a zeitgeber for the circadian clock [41]. LOV proteins in our data were associated with a range of effector domains, including kinases and b-ZIP transcription factors. LOV domains have rarely been characterized in marine plankton [42] and here demonstrated clear activity peaking just before dawn (Fig. S14).

### Timing of diel expression differed across taxonomic groups

Significantly periodic ORFs were classified into four bins based on the time of day of peak expression (Fig. 3). Most photosynthetic eukaryotes had periodic expression in all four bins, but the majority of expression from diel ORFs occurred in the day (Fig. 3c). Ciliates and other largely heterotrophic eukaryotes, on the other hand, had evening-dominated periodic expression [43]. For prokaryotes, the results were more mixed (Supplementary File 1).

### Timing of diel expression was partitioned by function

Across taxa groups, periodic non-organellar ORFs most commonly peaked in early day (~11 a.m.) and early night (~11 p.m.; Fig. 3d). Morning-peaking ORFs, coincident with peak photosynthetically active radiation, were dominated by photosynthesis and carbohydrate and lipid metabolism annotations. Evening-peaking ORFs related to chromatin structure and dynamics, cytoskeleton, and chromosome partitioning, possibly because evening-timed cell division minimizes UV-stress to an exposed genome [44, 45]. Comparative transcriptomics on *Ostreococcus*, *Chlamydomonas*, and *Arabidopsis* grown under alternating light:dark periods corroborates this conserved temporal partitioning of photosynthesis and cell-cycle genes [46].

WGCNA on all periodic ORFs independently recreated expression modules peaking in the early day, late day, early night, and late night (Fig. 3b). The early day module was most abundant, and was dominated by photosynthetic reaction center and chlorophyll AB binding proteins largely expressed by haptophytes, centric diatoms, pelagophytes, and some *Synechococcus* and *Ostreococcus* (Fig. 3e; red). This is consistent with laboratory findings for *Ostreococcus* [47] and vascular plants [38, 48] where mean peak expression of light harvesting and photosynthesis genes occurs in the middle of the day. Because our data is compositional in nature, it is a useful positive control to corroborate these established results with both WGCNA and HRA. When sampled in constant light (after a brief entrainment to 12:12-h light:dark cycles), many *Arabidopsis* nuclear-encoded photosynthesis genes also peak at “midday” [38], suggesting that the conserved midday peak we observe is circadian in nature. The high-turnover nature of the photosynthetic reaction center protein pool [49] makes it likely that the 11 a.m. peak expression of these transcripts correctly captures the timing of protein activity. The morning peak also contained a large number of transcripts for rubisco and ATP synthase, suggesting that carbon fixation and energy production are similarly timed. Chlorophyll synthesis ORFs (e.g., CobN/magnesium chelatase and geranyl reductase) also peaked at 11 a.m. across taxa groups, except in Proteobacteria, where bacteriochlorophyll synthesis peaked around midnight (Fig. S15c). Metabolism-related ORFs, including various ATP synthases, mitochondrial carrier proteins, phosphogylceride kinase, fructose-biphosphate aldolase, and fatty acid desaturase shared this mid-morning peak, but a second set of ATP synthases and mitochondrial carrier protein ORFs peaked in the early night coincident with cytochrome C oxidase and NADH-ubiquinone/plastoquinone oxidoreductase (Fig. S16a).

The early night module (Fig. 3e; green) contained an abundance of histones, especially from haptophytes and pelagophytes. DNA polymerases, condensins, cyclins, and CDKs also peaked around 11 p.m. across several photosynthetic eukaryotes (Fig. S15b). Synchronized populations of *Synechococcus* [50], *Ostreococcus* [47], *Chlamydomonas* [51], *Phaeodactylum* [6], *Emiliania* [52], and *Pelagomonas* [53] divide in early night when grown on a 12:12-h light:dark cycle, consistent with the cell division machinery we observed peaking at night in *Synechococcus*, *Ostrococcus*, prasinophytes, diatoms, haptophytes, pelagophytes, and other eukaryotes. Axonemal ORFs were significantly periodic across a broad range of motile taxa, including pelagophytes (14 ORFs), ciliates (2 ORFs), haptophytes (1 ORF), and chlorophytes (1 ORF), all peaking in the early evening (mean ~9 p.m.).

Translation-related ORFs were also periodic across many lineages. Translation initiation factors were periodically transcribed in centric diatoms, ciliates, haptophytes, other chlorophytes, pelagophytes, and *Synechococcus* (Fig. S16d). Interestingly, we identified several periodic eukaryotic translation elongation factor 3 (eEF3) ab initio ORFs in the large fraction (Fig. S16d). Previously believed to be unique to fungi, eEF3 presents a novel peptide synthesis mechanism for phytoplankton.

### Physiological interpretation of diel transcriptional partitioning

For high-turnover proteins, diel transcription is likely important for maintaining appropriate protein levels. However, many protein pools turnover too slowly for diel transcription to translate into diel changes in protein abundance, which is determined not only by transcription rates, but also translation and degradation rates. In *Arabidopsis*, *Ostreococcus*, and *Cyanothece*, the majority of proteins have half-lives that span multiple diel cycles [54]. In *S. elongatus*, only about 5% of proteins exhibit the diel dynamics that 30–60% of transcripts do [54, 55]. In *O. tauri*, under 10% of proteins are rhythmic despite nearly the whole transcriptome oscillating [56]. In this low-turnover case, diel transcriptional rhythms are more difficult to interpret.

**Fig. 4:**
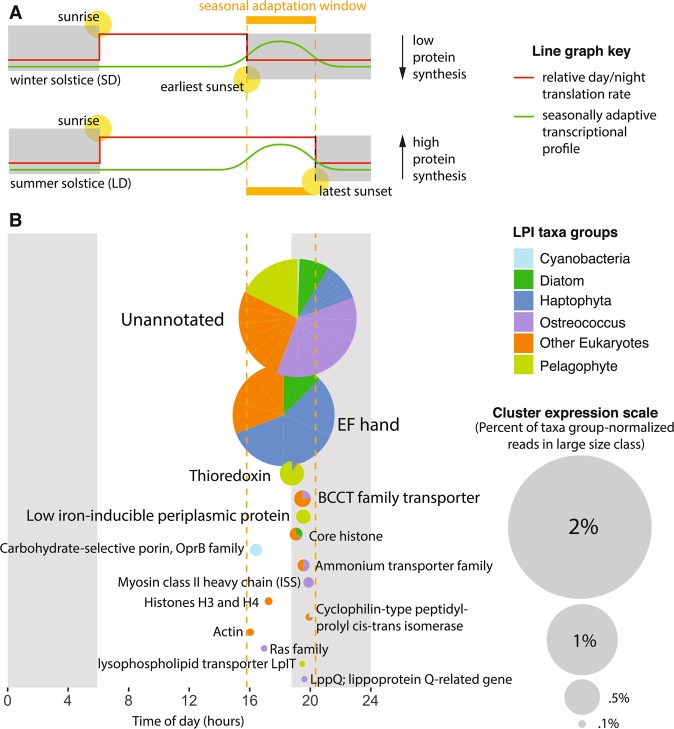
Translational coincidence as a mechanism for seasonal adaptation in algae. **a** Schematic adapted from Seaton et al. [54] depicting the translational coincidence mechanism. The top graphic represents the shortest photoperiod of the year, the winter solstice (December 21, 2010); the bottom graphic represents the longest photoperiod of the year, the summer solstice (June 21, 2010). An ORF (green line) peaking in the early night during short day (SD) conditions (top) would peak in the late day during long day (LD) conditions (bottom). Because the average translation rate (red line) is much higher during daylight hours than night hours (gray boxes), an ORF peaking in this “seasonal adaptation window” (orange) would be upregulated in LD at the protein level. **b** Top non-organellar cluster annotations from the large size class of our drift track that are significantly diel and peak in the seasonal adaptation window (orange dashed lines). Pies are colored by relative taxonomic contribution and scaled to reflect proportional transcript abundance (legends, right)

One explanation for diel cycling transcripts in the case of stable protein abundance is “translational coincidence”, a mechanism described in *Arabidopsis* in which the timing of transcription and translation interact to optimize use of solar energy for a given photoperiod [54]. In photosynthetic organisms spanning cyanobacteria, chlorophyte, and plant lineages, protein synthesis rates are 3–5 times higher during day than night [54]. In a dawn-tracking circadian clock, transcripts that peak in the late day during a long photoperiod (e.g., summer) peak after sunset during a short photoperiod (e.g., winter; Fig. 4a). Proteins with late-day transcripts would therefore be more abundant in long photoperiods because of reduced translation rates after sunset. In contrast, transcripts peaking in the early day or late night would not have seasonally variable protein pools. This mechanism is not only highly relevant for plants, in which seasonal adaptations spanning diverse physiological mechanisms from flowering to freezing tolerance are well described [54], but are also likely critical for unicellular algae. Seasonal phenotypes are poorly described in algae due to difficulty of observation, but some examples have been reported. For example, in response to short photoperiods (e.g., winter) *Lingulodinium* forms cysts and *Chlamydomonas* suppresses zygospore germination [57].

To assess what functions might be influenced by such seasonal adaptation, we analyzed 276 significantly periodic nuclear ORFs that peaked within the window in which seasonal adaptation would be expected (9.9–14.4 h after local dawn; Fig. 4b). All taxa groups that showed significantly periodic activity had ORFs peaking in this window except dinophyta and “other Proteobacteria”. In the cCCS transition zone, iron limitation increases in tandem with photoperiod as the upwelling season progresses from spring into late summer [21]. Because iron is a photosynthetic cofactor, the stress of increased day length and low-iron likely compound in this season.

Indeed, the top annotations peaking in the seasonally adaptive window are implicated in responding to low iron and UV-stress. The most highly expressed annotation was the calcium-binding domain, EF-hand. Calcium signaling is best known in algae as being required for photoacclimation [58] and low-iron response [59]. EF-hand-containing proteins, specifically, are associated with the low-iron phenotype in diatoms and haptophytes [8, 60]. The second most abundant annotation, thioredoxin, modulates the activity of photosynthesis proteins in response to light by sensing redox potential [61]. Three thioredoxins were significantly upregulated during long photoperiods in the *Arabidopsis* proteome [54]. Finally, low-iron-inducible periplasmic protein was the fourth most abundant annotation, and was dominated by the iron-uptake protein ISIP2A (phytotransferrin; ref. [62]).

When viewed with WGCNA, ISIP2A expression clustered with silicon transporters (Fig. S5, module 5; Supplementary File 1). Both were chiefly expressed by centric diatoms, which may be more sensitive to iron stress [63] because they tend not to use the ferritin mechanism favored by bloom-forming pennates. The expression pattern of module 5 had some day/night signal, but also peaked strikingly at the end of the drift track, when the silica:nitrate ratio dropped most dramatically. Low silica:nitrate ratios have been observed in association with iron limitation [20] and are thought to result from silica drawdown by iron-stressed diatoms [23]. This convolution of the influence of day/night cycles and nutrient limitation on patterns of expression is indicative of how transcription may be responding to multiple drivers in a dynamic natural context. Whereas the “up-at-dusk” component of module 5 expression could be a circadian-driven mechanism allowing the phytoplankton to be generally more responsive to iron stress in the season when it is most exacerbated, the apparent additional level of upregulation on day three is likely a response to local conditions—namely, increasing iron stress at the end of the drift.

### Cascade of photosynthetic activity takes place in the morning

**Fig. 5:**
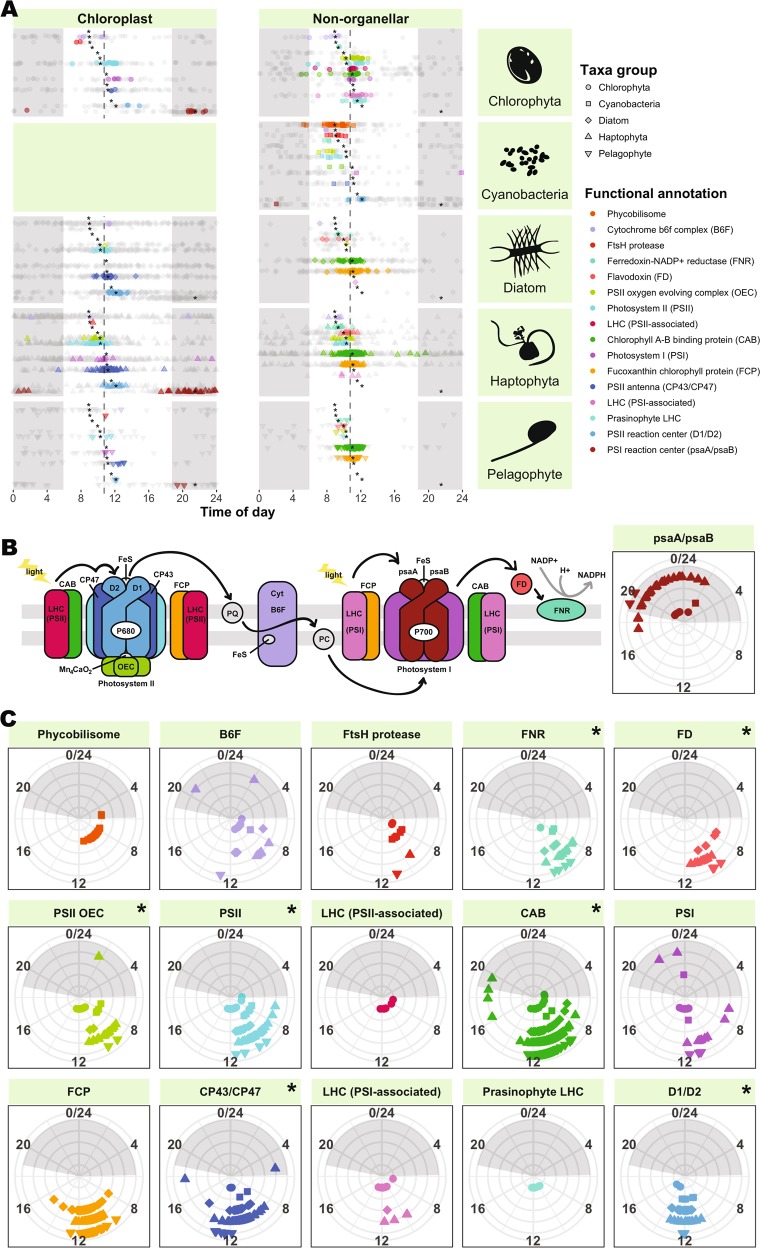
Peak expression time of large fraction ab initio ORFs involved in photosynthesis. Night is indicated by gray shading. **a** Cascade of peak expression time occurs across diverse phytoplankton lineages. ORFs are plotted by chloroplast encoded (left) and non-organellar (right). Significantly periodic ORFs (HRA; FDR ≤0.1) are colored by functional annotation (legend, right) and plotted in the same order as shown in the legend; insignificant ORFs are shown in grey. Asterisks denote functional annotation means across taxa groups that differ significantly (Watson–Wheeler Test of homogeneity of means, averaged over 1000 iterations to break ties, and Benjamini–Hochberg FDR <0.05) from the overall mean peak expression time (dashed gray line; 10:53 a.m.). **b** Illustration of the photosynthetic apparatus, colored by functional annotations from parts (**a**, **c**). Components with fewer than ten significantly periodic ORFs (pastoquinone (PQ), plastocyanin (PC)) are depicted in gray. Black arrows denote electron transport. **c** Conservation of peak expression time across phytoplankton lineages. Taxa groups are distinguished by shape (legend, top right) and radius (innermost: chlorophyta, outermost: pelagophyte). A Watson–Williams Test of homogeneity of means was performed on each functional group to determine taxonomic differences in peak expression time. Annotations with significantly different peak expression time across taxa groups (Benjamini–Hochberg FDR <0.05) are indicated with an asterisk

In contrast to the long-lived proteins whose diel transcription may be seasonally relevant, short-lived proteins with diel oscillating mRNA are likely of daily importance. In our data, periodic photosynthesis ORFs peaked at a mean of 10:53 a.m. based on a sinusoidal fit (dashed line; Fig. 5a), but components of the photosynthetic apparatus peaked in a “cascade” throughout the morning, beginning around 9 a.m. with the phycobilisome (orange) and cytochrome b6f complex (lavender), and ending around noon with PSII reaction center D1/D2 (blue). *ftsH* protease was also periodic and peaked in time with photosynthesis genes in cyanobacteria and in eukaryotes. This is likely due to its role in both cyanobacteria [64] and in eukaryotic chloroplasts [49] in repairing PSII via the degradation of D1 proteins damaged by oxidative stress. All photosynthesis protein categories except PSI (violet) and light harvesting complexes associated with PSI (pink) and PSII (fuchsia) were significantly different from the overall photosynthesis mean peak time of 10:53 a.m. (Watson–Wheeler Test of Homogeneity of Means, FDR <0.05).

The timing of most of our photosynthesis ORFs is consistent with previous findings for naturally occurring picoplankton assemblages (Fig. S17). Similar photosynthetic cascades have also been observed in algae grown in light:dark conditions. In *Micromonas pusilla*, photosynthesis components show pronounced transcriptional changes in connection with the transition away from dawn [65]. In a higher resolution study of *Chlamydomonas* [51], patterns similar to those observed here were observed, but shifted slightly later, with B6F peaking early in the subjective day (~ZT4; 10 a.m.), followed by LHCI, PSI, and PSII in the middle of the subjective day (~ZT7; 1 p.m.), and LHCII last (ZT8; 2 p.m.). Likewise, in the diatom, *Phaeodactylum*, light harvesting machinery also peaks in the late afternoon [6]. This relative delay could be an effect of comparing 12:12-h light:dark cycles with natural conditions where daylight persists longer than 12 h.

Surprisingly, PSI reaction center ORFs, *psaA* and *psaB*, consistently peaked at night (~9:30 p.m.) in cyanobacteria and the chloroplasts of chlorophytes, haptophytes, and pelagophytes (Fig. 5; dark red), rather than forming part of the daytime expression cascade. This runs contrary to previous findings for *Prochlorococcus*, where *psaA* and *psaB* have been shown to peak around 8 a.m. in environmental [11] and noon in laboratory [66] settings. This difference could possibly be explained by the iron-limited setting of our drift track. Iron-rich components of PSI are downregulated during iron stress in *Prochlorococcus* [67], and *psaA* expression is specifically controlled by an Fe-responsive regulator in *Chlamydomona*s [68]. Deviations from the midday photosynthetic cascade also occurred in PSI and B6F, the two electron transport chain components that bind the most iron–sulfur clusters and are iron-stress responsive in diatoms [69]. However, to the best of our knowledge, such a dramatic shift in expression has not been previously been observed for *psaA*/*psaB* and its cause is ultimately unknown.

### Diel transcriptional patterns vary across taxa

The percent of the overall transcriptome with significant 24-h oscillations varied greatly between taxonomic groups, and was highest in *Ostreococcus* (nearly 25%) and lowest in dinoflagellates (<1%; Fig. 3a). This difference could be explained by varied biological mechanisms for responding to sunlight. For example, dinoflagellates [70, 71] and ciliates [72] likely rely on post-transcriptional or post-translational mechanisms to a great degree. Here, dinoflagellates exhibited relatively consistent expression profiles that were anomalously highly correlated for eukaryotes (*r* = 0.57; Fig. S11a).

Dinoflagellates have been suggested to regulate their transcription differently than other algae and primarily respond to the environment post-transcriptionally [71, 73–77]. Dinoflagellate circadian oscillations have usually been observed at the level of translation [78, 79], with reports of only 3% of *Pyrocystis lunula* genes being transcribed on a day/night cycle [70] and even circadian-controlled cell-cycle regulators being post-transcriptionally controlled in *Karenia brevis* [71]. In *Lingulodinium polyedrum*, iron superoxide dismutase protein levels peak at midday, despite arrhythmic mRNA [73].

Despite their post-transcriptional response strategy, we detected 13 significantly periodic dinoflagellate nuclear ORFs, all of which recruited at least 69 reads. Eleven peaked in the early day and consisted of photosynthesis-related ORFs (chlorophyll A–B binding proteins or GAPDH), hypothetical proteins, and ATP sulfurylase, which catalyzes the first step in sulfate assimilation [80]. The remaining two ORFs both peaked around 1 a.m. and consisted of karyopherin alpha, an adaptor protein responsible for importing proteins to the nucleus, and a small ubiquitin-related modifier protein involved in post-translational modification. Diel cycling of such protein modifiers could shed light on currently cryptic diel behavior, such as bioluminescence, in dinoflagellates.

In contrast to the dinoflagellate and ciliate results, the high percentage of periodic ORFs in other taxa could be explained by light-synchronized division (as in *Ostreococcus*), and cyclic expression of transcriptional machinery such as the preinitiation complex, RNA polymerase, and transcription factors in prasinophytes, *Synechococcus*, haptophytes, and pelagophytes (Fig. S16c).

In our data and the literature, diel partitioning of large portions of the transcriptome is a strategy most often adopted by phytoplankton with small cells and fast division rates. Diel transcription could allow such “streamlined” organisms to synchronize their protein pool with their transcript pool without manufacturing a large number of ribosomes. Temporally segregating a given transcript would increase its proportional abundance at the time of translation, which, according to information theory [81], could decrease fluctuations in protein number caused by ribosome sampling stochasticity. This could also provide an evolutionary motivation for producing precise waves of transcription for proteins that do not oscillate on a day/night cycle, as has been the perplexing case for the majority of genes in *S. elongatus* [54, 55] and *O. tauri* [56]. In contrast, we may observe fewer cyclic transcripts in larger organisms (e.g., ciliates, dinoflagellates) because they have the resources to maintain large transcript pools across all times of day. Indeed, large transcript pools could also provide greater flexibility in response to sudden environmental change, which may benefit the heterotrophic capabilities of these large taxa.

### Host-virus interactions

**Fig. 6:**
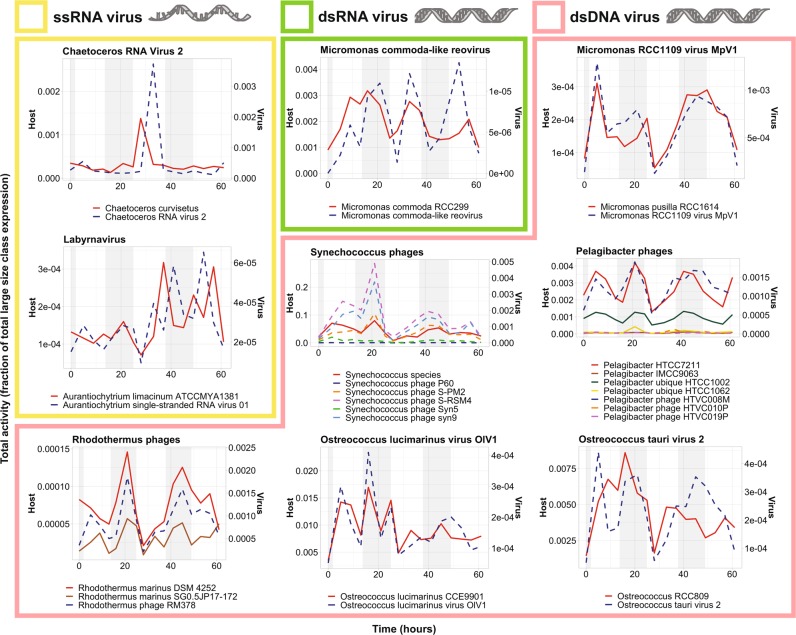
Virus/host dynamics in the large size class. Viruses and hosts are annotated as the closest reference available in our database, as determined by LPI. Library normalized expression of ORFs classified as ssRNA (yellow), dsRNA (green), and dsDNA (pink) viruses and their putative hosts by LPI are shown. Putative host expression is represented by solid lines and corresponds to left y-axes; virus expression is represented by dashed lines and corresponds to the right y-axes. Night hours are shaded in gray. Note that OtV2 infects RCC393, an *Ostreococcus* Clade OII species, not *O. tauri*. OtV2 was isolated against *Ostreococcus* Clade OII isolate RCC393 [94] which has 99% 18S rDNA identity to the genome sequenced Clade OII isolate RCC809 used in our mapping analysis. Likewise, the *M. commoda-like* reovirus infects the strain LAC38, which was initially misreported as being *M. pusilla* and has been renamed here according to proper species assignment of the host [87]. Interestingly, while picoprasinophyte populations mapping most closely to *Micromonas pusilla* were coactive with a dsDNA virus, populations mapping most closely to *Micromonas commoda* (RCC299) were coactive with a dsRNA virus [87, 95]

We observed a diversity of viruses infecting bacteria and eukaryotes in both size classes (Fig. 6, S18). Viruses infecting large phytoplankton (e.g., *Heterocapsa*, *Chaetoceros*, *Emiliania*, and *Phaeocystis*) were enriched in the large fraction (Supplementary Dataset 10), whereas, in the small fraction, bacteriophages corresponding to several of the abundant bacterial groups were enriched (e.g. *Pelagibacter* phage, log_2_FC = 11; *Roseobacter* phage, log_2_FC = 7.4; *Vibrio* phage, log_2_FC = 5.2; Supplementary Dataset 10; Supplementary File 1).

In the large size class, the majority of viruses were dsDNA viruses (74%), but we also observed RNA viruses that infect phytoplankton [82] and labyrinthulids. While RNA virus reads could represent either transcribed mRNA or RNA genomic material, DNA virus transcripts indicate an active infection.

Remarkably, rather than host and virus expression being anticorrelated, as one might expect from kill-the-winner theory [83], co-expression was observed between dsDNA viruses and their hosts, which were homologous to diverse reference taxa including heterotrophic bacteria (e.g., *Pelagibacter*, *Enterobacteria*), cyanobacteria (e.g., *Synechococcus*, *Prochlorococcus*), photosynthetic eukaryotes (e.g., *Bathycoccus*, *Micromonas, Ostreococus lucimarinus*), and predatory heterotrophic eukaryotes (e.g., *Cafeteria roenbergensis*; Fig. 6, S18; pink boxes). One exception was *Phaeocystis*, with its aggregate gene expression peaking during daylight hours while both the giant *Phaeocystis globosa* virus and its virophage peaked synchronously at night. In addition, a virus infecting *Ostreococcus* Clade OII (OtV2) had clear night peaks in transcription, a phenomenon that has only ever been observed in the laboratory in experiments with the most distant of other *Ostreococcus* species, *O. tauri*, when infected by OtV5 [84]. Cyanophages also had peak expression at night, as previously observed [12]. The coordinated expression we observe between dsDNA viruses and hosts may result from replication of large viruses being more demanding on host metabolism [85, 86]. However, because we sequence bulk populations, we cannot be certain that host and virus transcripts originate from the same cell.

Unlike the predominance of host:dsDNA virus co-expression, ssRNA viruses related to those infecting the diatom, *Chaetoceros*, and the labyrinthulid, *Aurantiochytrium* were not co-expressed with their putative hosts. Rather, virus RNA molecule abundance lagged behind host transcription (Fig. 6; yellow boxes).

Most of the reference viruses that were used for gene mapping in our analyses have been well characterized. In laboratory experiments, it has been shown that their lytic cycles are 24–48 h in length even under various forms of nutrient limitation [82, 84, 87–89]. Hence, the detection of daily, closely synchronized transcription over more than two day/night periods suggests that a subset of each population of the major microbial players in this system, whether photosynthetic or not, was infected and lysed multiple times during the time course. Thus, rather than a few taxa being in a bloom scenario with epidemiology that would facilitate a massive viral lysis event, most taxa appeared to be under perpetual predation by viruses.

### Conclusions and future directions

We present results from an often iron-limited, high-nutrient, low-chlorophyll setting in the eastern north Pacific [21]. We observed a pronounced transcriptional response indicative of existing iron limitation as well as a mechanism for seasonal adaptation to low iron and long photoperiod. We demonstrate that future measurements of diel transcription have the potential to elucidate not just daily, but also seasonal, phytoplankton physiology by considering the implications of varied day/night translation rates. Due to its high resolution and semi-Lagrangian nature, samples collected from this drift track present an opportunity to study in situ phytoplankton physiological responses to day/night cycling—something rarely investigated for eukaryotes. We report, for the first time in nature, diel cycling transcription across major phytoplankton lineages. The proportion of genes cycling varied taxonomically, possibly reflecting differences in life strategy or post-transcriptional regulation.

Notably, laboratory observations of marine phytoplankton (e.g., *Thalassiosira* [90], *Ostreococcus* [47], *Synechococcus* [91], and *Prochlorococcus* [66]) report larger percentages of the transcriptome oscillating than we observed. This is likely due to increased statistical power enabled by deeper sequencing and higher percentages of mapped reads (i.e., mapping to a single model genome) as well as greater replication [92], but may also reflect physiological differences between ideal growth conditions and the patchy, dynamic nature of the marine environment.

Regardless of the proportion of genes cycling, functional partitioning of cycling genes was common across phytoplankton, pointing to a shared need to prepare for the daily onset of solar radiation and partition metabolic activity accordingly. Whereas heterotrophic bacterioplankton have been observed to display consecutive peaks in translation and oxidative phosphorylation-related transcription throughout the day [13], phytoplankton showed largely synchronized transcriptional timing. This may reflect differences between heterotrophic and photosynthetic life strategies. The unique metabolic needs of one bacterioplankton species might allow it to benefit from temporal association with another, but the largest driver of phytoplankton energetics is solar energy, which is concurrently available to all taxa. Our observation of a taxonomically conserved cascade of photosynthetic activity centered around ~11 a.m. and relegation of cell division to night [44, 45] may be explained by peak solar radiation and the risk of oxidative stress: both selective pressures that are shared by all photosynthetic organisms.

In addition to abiotic stressors, diverse phytoplankton and bacterial taxa appeared to be coping with viral infections, evidenced by high levels of viral RNA that often tracked putative host expression patterns. Indeed, the short lytic cycle of some of the closest viral references indicates that many host populations likely experienced multiple consecutive cycles of growth and viral lysis within the ~2.6 -day drift.

To date, in situ molecular sampling of large phytoplankton in the environment has not been applied widely, but future drifts have the potential to tease apart drivers of productivity in varied ocean conditions. Our results highlight the diversity of uncultured oceanic phytoplankton who remain “microbial dark matter” and the complexities of biogeochemical cycling by community interactions that are yet to be elucidated. We demonstrate that this technology allows for the observation of phytoplankton communities within the context of their natural biotic interactions and dynamic abiotic stressors. Such contextualization is an important step towards ascertaining global phytoplankton resilience to perturbation, and in turn, the resilience of the ecosystem services they provide.

## Supplementary information

S1: Supplemental Text

S2: Supplemental figures

S3: Description of supplemental datasets

Supplemental Dataset 1

Supplemental Dataset 2

Supplemental Dataset 3

Supplemental Dataset 4

Supplemental Dataset 5

Supplemental Dataset 6

Supplemental Dataset 7

Supplemental Dataset 8

Supplemental Dataset 9

Supplemental Dataset 10

Supplemental Dataset 11

Supplemental Dataset 12
