# Supplementary material for: Diel transcriptional response of a California Current plankton microbiome to light, low iron, and enduring viral infection
Source: ISME J. 2019 Jul 18;13(11):2817–33. doi: 10.1038/s41396-019-0472-2 (PMC6794264; doi:10.1038/s41396-019-0472-2)
Supplement: Supplementary file 1 — S1: Supplemental Text [file 41396_2019_472_MOESM1_ESM.pdf]

Supplementary File 1: Supplementary Text

Supplementary Materials and Methods

Sample Collection

Samples were collected in association with Ottesen et al. 2013 off the coast of California from September 16-19, 2010 along the warm side of an upwelling-driven front (1). Briefly, the Environmental Sample Processor (ESP; 2) was suspended at 23 m depth below a semi-Lagrangian surface float, collecting 1L of seawater every ~4 h for 61 h (~2.6 days). Retained particulates were size fractionated onto 5 μm and 0.22 μm Durapore 25 mm filters (Millipore, Billerica, MA, USA), preserved immediately *in situ* via a 2 min incubation in RNALater (Ambion) and stored at -80°C within 36 hours of ESP recovery. Nutrients, chlorophyll, and other oceanographic metadata was obtained via shipboard CTD/niskin rosette casts (Supplementary Dataset 12) as previously described (3). Drift speed was determined via a surface-float mounted GPS sensor. Water speed relative to the drifter was measured using a surface-float mounted ADCP (Supplementary Dataset 12A) in order to detect deviations from truly Lagrangian sampling (e.g. wind forcing).

Metatranscriptome library preparation and sequencing

Small size class metatranscriptomes were sequenced by Ottesen et al. using a GS Titanium system (Roche) according to their previously published methods (1). Large size class cDNA was prepared as in Ottesen et al. from ribosomal RNA-depleted

total RNA (1). 1 ul of cDNA per sample was used to prepare metatranscriptome libraries with the Truseq RNA Sample Prep kit v2 (Illumina™) according to manufacturer's instructions starting from the end repair step. Libraries were paired-end sequenced on the Illumina HiSeq 2000 platform to obtain 2x100bp reads.

Amplicon library preparation and sequencing

Large size class 16S and 18S ribosomal RNA were sequenced using 454 GS FLX Titanium pyrosequencing. Nearly universal bacterial primers 341F (5'-CCTACGGGNGGCWGCAG-3') (4) and 926R (5'-CCGTCAATTCMTTTRAGT-3')(5) were used to target the v3v5 region of 16S and primers and TAReuk454FWD1 (5′-CCAGCASCYGCGGTAATTCC-3′) and TAReukREV3 (5′-ACTTTCGTTCTTGATYRA-3′) (6), were used to target the v4 region of 18S, each amplifying an approximately 500 bp region of cDNA. FLX Titanium adapters (A adapter sequence: 5′ 127 CCATCTCATCCCTGCGTGTCTCCGACTCAG 3′; B adapter sequence: 5′ 128 CCTATCCCCTGTGTGCCTTGGCAGTCTCAG 3′) and 10bp multiplex identifier (MID) barcodes were used for multiplexed 454 sequencing.

cDNA was prepared from 50 ng per sample of total RNA using the Life Technologies SuperScript III First Strand Synthesis system with random hexamer primers. cDNA concentration ranged from 312 – 18,440 pg/microliter. 1 µl of cDNA was used as a template amplified using Life Technologies AccuPrime PCR system kit, in a reaction containing 1X AccuPrime Buffer II, .75 units of AccuPrime Taq High Fidelity, and a final primer concentration of 200 nM, alongside a no template negative control for cDNA synthesis. Amplifications were performed using a Life Technologies ProFlex PCR system, with an initial denaturation at 95°C for 2 minutes, 30 cycles of 95°C for 20

seconds, 56°C for 30 seconds, 72°C for 5 minutes. PCR products (2 µl of each sample and 5 µl of negative control) were run on a 1% agarose gel at 105 V for 35 minutes, then cleaned with Ampure XP beads (Beckman Coulter, Brea CA), and resuspended in 25 µL of Qiagen elution buffer. 2.5 µL was used for visualization on an agarose gel, 1 µL was used in a LifeTechnologies' PicoGreen Quant-IT assay to quantify the final product, and 45 ng of both 16S and 18S amplicons were pooled separately for 454 pyrosequencing.

The vendor's standard protocols (Roche Diagnostics) were used for library QC, emPCR, enrichment and 454 sequencing with the following modifications: KAPA Biosystems Library Quantification Kit for qPCR was used to accurately estimate the number of molecules needed for emPCR, automation (BioMek FX) was used to "break" the emulsions after emPCR, and butanol was used to for ease of handling during the breaking process. The bead enrichment process was automated by using Roche's REM e (Robotic Enrichment Module).

Bioinformatic analysis of metatranscriptomes

See Figure S2 for illustration of metatranscriptomic analysis pipeline.

Open reading frame (ORF) calling and annotation

Large fraction Illumina reads and small fraction 454 reads were processed via the RNAseq Annotation Pipeline (rap) v0.4 (7). Small fraction reads were obtained via DBCLS SRA (http://sra.dbcls.jp/) using accession number SRA062433. Reads from both fractions were trimmed to remove primers and areas of low sequence quality (reads must be at least 30 base pairs (bp) long and have a quality score of at least 33 to

be retained). Illumina reads were paired. Ribosomal RNA (rRNA) reads were removed using Ribopicker v.0.4.3 (8). Large fraction reads were assembled using CLC Genomics Workbench 9.5.3 (https://www.qiagenbioinformatics.com/) first by library, then overall. Small fraction reads were left unassembled due to the longer read length, lower coverage nature of 454 sequencing. *Ab initio* ORF prediction was performed with FragGeneScan v1.16 (9) with parameters: complete=0 and train=complete. ORFs were once again screened for contamination in the form of rRNA, ITS, and primers. ITS sequences were downloaded from NCBI, and reduced to 397,062 non-redundant sequences at 0.95 level using cd-hit-est v4.6. Sequences that aligned with an ITS sequence with BLASTN e-value <= 1e-5 were removed. Primer and adapter sequences used in Illumina sequencing were searched using BLASTN and were identified at e-value <= 10. Reads were removed with hits to terminal ends at least 10bp in length, or internal hits at least 15bp in length. Possible organelle genes were classified for query sequences that had closer homology to an organelle gene than a nuclear gene within the organism with the closest known segregated organelle and nuclear genomes based on best BLASTP e-value <= 1e-3.

ORFs were annotated via BLASTP (10,11) alignment (e-value threshold $1e^{-3}$) to a comprehensive protein database, *phyloDB*, as well as screened for function *de novo* by assigning Pfams, TIGRfams and transmembrane tmHMMs with hmmer 3.0 (http://hmmer.org/; 12) using an e-value threshold of $1.0e^{-4}$. PhyloDB version 1.076 consists of 24,509,327 peptides from 19,962 viral, 230 archaeal, 4910 bacterial, and 894 eukaryotic taxa (13–15). It includes peptides from the 410 taxa of the Marine Microbial Eukaryotic Transcriptome Sequencing Project

(http://marinemicroeukaryotes.org/), as well as peptides from KEGG, GenBank, JGI, ENSEMBL, CAMERA to KEGG, GenBank, JGI, ENSEMBL, iMicrobe, and the Chloroplast Genome Database (cpbase). Taxonomic annotation of ORFs was also conducted via a BLASTP to phyloDB, and a Lineage Probability Index (LPI) was calculated to avoid biases introduced by classifying ORFs based on best BLAST hit alone (7,16,17). Briefly, LPI was calculated here as a value between 0 and 1 indicating lineage commonality among the top 95-percentile of sequences based on BLAST bit-score.

Illumina sequencing of large size fraction total ribosomal-depleted RNA yielded 623,461,310 raw reads across 16 time points, of which 265,345,754 were mRNA (~43%). A total of 283,760 contigs were assembled upon which 345,355 ORFs were called. 32,271,421 reads (~12%) mapped to the 111,655 ORFs that remained after strict filtering (~32%). Ottesen et al.'s GS FLX Titanium (Roche) sequencing of small fraction cDNA yielded 9,985,281 raw reads across 13 time points (1). 2,802,084 ORFs were called on the 5,618,280 trimmed reads remaining after quality control (~56%). Full assembly and annotation statistics in Supplementary Dataset 1. Coverage across taxa groups can be seen in Figure S3A.

Mapping to reference transcriptomes

Reference transcriptomes were chosen for read mapping that appeared with high abundance and percent identity among *ab initio* large fraction ORFs. Representative references were chosen from all major taxonomic groups found in *ab initio* ORFs. Large and small fraction reads were aligned to reference ORFs using BWA-MEM

version 0.7.12-r1039 (18,19) using default parameters. At least 50% of each read must map to a reference gene at least 80% identity to be considered a hit. References with at least 1000 genes with at least 5 reads mapped were functionally annotated via rap v0.4 as above and considered for downstream analysis. Coverage of annotated references can be seen in Figure S3B.

Hierarchical clustering

Reference transcriptome ORFs were hierarchically clustered together with all large and small fraction *ab initio* ORFs (including organellar ORFs) to form peptide ortholog groups via the Markov Cluster Algorithm (MCL; https://micans.org/mcl/; 20). Directional edge weights were defined as the ratio of pairwise- to self- BLASTP scores, and default parameters were used to assign ORFs to clusters. Clusters were assigned a consensus annotation if found to be statistically enriched in that annotation with a Fisher's exact test ($p < 0.05$). Consensus annotations must also represent at least 10% of the reads in the cluster and account for a minimum of 200 reads. Clusters with identical consensus annotations were grouped together into "functional clusters."

Identification of significantly periodic ORFs

ORFs with significantly periodic diel expression were identified using harmonic regression analysis (HRA) as previously described (1,21,22). Briefly, for each taxa group of interest, raw ORF counts over time were fit to a generalized linear model (glm) of a sinusoid with a 24-hour period with taxa-specific library sums serving as an offset at each time point. The model was constructed using the "glm" function in R (23) and

statistical significance was determined by False Discovery Rate (FDR; (24) adjusted p-values of <= 0.1 (Benjamini-Hochberg), on both a permutation test (500-50,000 permutations) and a chi-squared test.

Identification of conserved expression modules

The Weighted Gene Correlation Network Analysis (WGCNA) R package (25,26) was used as previously described (22) to identify modules of conserved expression among reference ORFs (Figure S13) and functional clusters (Figures 2, 3E, S5). ORFs/functional clusters with at least 10 raw counts in at least 80% of time points were considered. For Figure 3E, it was further stipulated that clusters must have at least 100 raw reads overall to be considered. In all cases, expression was normalized by total pre-filtration counts at a single time point (library) before constructing a Pearson correlation matrix. An adjacency matrix was then constructed from the correlation matrix by applying a power function (AF(s)=s^b). The lowest b value that allowed for a scale-free topology R-squared value above 0.8 was chosen, as recommended in the WGCNA user manual, in order to optimize the mean number of connections of the network while preserving scale independence. A signed Topological Overlap Matrix (TOM) was constructed from the correlation matrix to measure dissimilarity between each pair of nodes based on shared neighbors. Average linkage hierarchical clustering was used to define a dendrogram (cluster tree) of the network via the “blockwiseModules” function. A cut height of .995 as well as a minimum module size of 30 ORFs/functional clusters was used to delineate branches of the hierarchical clustering tree into modules of co-expression. The “moduleEigengenes” function was used with default parameters to

calculate “eigengenes” (a measure of “average” expression calculated as the first principal component of the module's expression matrix) for each module. Modules with correlated eigengenes were merged by setting a “mergeCutHeight” threshold of 0.5. ORFs/functional clusters with a correlation of less than 0.3 to their respective module eigengene were removed and classified as “unassigned” (module 0). The igraph package (27) was used to visualize expression networks.

Differential expression analysis

Differential expression of ORFs, ortholog clusters, taxa groups, and genera across size classes was identified using the R package edgeR (28). Categories (i.e. ORFs, ortholog clusters, taxa groups, genera) with least 1 read per million in at least 3 samples were included and used to calculate log fold changes. Counts were normalized using the “calcNormFactors” function, which accounts for both library size and varied library composition. An exact test with tagwise dispersion estimation was used to determine ORFs or clusters with significantly different expression across size classes (FDR-corrected $p < 0.05$).

Identification and phylogenetic analyses of LOV domain containing transcripts

A two-step approach was taken to identify LOV domain containing proteins in our reference and *ab initio* transcript sets. LOV domains are a subset of the PAS domain family, and initial survey of a number of known LOV domain proteins using InterproScan (29) suggested the PAS_9 Pfam domain (PF13426) has the highest similarity to the LOV domain. For this reason, we curated a list of transcripts harboring the PAS_9 domain

(detail of domain annotation is provided in the 'Bioinformatics analysis of metatranscriptomes' section). LOV domains have a signature motif that has a conserved cysteine at the fourth position, however, some degeneracy can exist at other positions of this domain (30). Given this fact, we further screened the amino acid sequences of the transcripts harboring the PAS_9 domain for the presence of previously-identified LOV specific motifs (30). We constructed a maximum likelihood phylogenetic tree from only the regions of the proteins that aligned to the PAS_9 HMM. Alignment was performed using MUSCLE (31). The tree was constructed in PhyML (32) with aLRT-SH like node support. The tree and the heatmap of the expression profile were visualized in the interactive Tree of Life (33).

Analyses of circular "time of day" data

The R package "circular" (34) was used to conduct circular statistics on time-of-day data with a 0-24 hour range. This includes peak time of day comparisons, calculating the mean peak time of expression of a group of periodic ORFs, and statistics on the photosynthetic cascade (Figure 5): Watson-Wheeler Test of homogeneity of means, Watson-Williams Test of homogeneity of means (35).

Figures

Sorting and plotting of data was conducted in R version 3.2.1 (2015-06-18) using the following packages: plyr (36), dplyr (37), reshape2 (38), ggplot2 (39), lubridate (40), ggmap (41), gridExtra (42).

Bioinformatic analysis of amplicon data

rRNA read processing and annotation

16S and 18S rRNA 454 reads were demultiplexed using Roche/454's sfffile utility and converted from standard flowgram to fasta format using sff2fastq (https://github.com/indraniel/sff2fastq). Primer removal, quality control, trimming, dereplication, and taxonomic annotation were conducted using an in-house rRNA pipeline (https://github.com/allenlab/rRNA_pipeline). Chimeric sequences were removed using USEARCH (43), reads were trimmed to a quality score of 10 over a 2 base window, operational taxonomic units were clustered using SWARM (44) and classified using FASTA36 from the FASTA package (http://faculty.virginia.edu/wrpearson/fasta/fasta36/). Taxonomic annotations were assigned by using GLSEARCH36 (45) with the version 119 of the SILVA reference database (46) for 16S rRNA and a modified PR2 database with updates from Tara Oceans W2 (47) for 18S rRNA.

Roche 454 sequencing of large fraction amplicons across all 16 time points yielded 820,700 raw 16S rRNA reads and 970,927 raw 18S rRNA reads, of which 36.7% (301,244) and 38.7% (375,904), respectively, remained after filtration. After de-replication, the large fraction contained 8,522 unique 18S and 5,420 unique 16S reads (1,595 of which were plastid in origin). Full pipeline statistics shown in Supplementary Dataset 2.

Phylogenetic placement

rRNA amplicons were processed against rRNA reference covariance models using Infernal (48). A blastn (11) search was performed against SILVA (46) with *e*-value threshold ≤ 1E-100 to identify representative (reference) sequences to be included in the reference phylogenetic trees (eukaryotic 18S, bacterial 16S, and plastidic 16S). Reference sequences were then aligned with MAFFT (49) using the G-INS-i setting for global homology. The generated multiple sequence alignments were visually inspected, manually edited and refined using JalView (50). Maximum likelihood reference trees were inferred under the general time-reversible model with gamma-distributed rate heterogeneity using FastTree (51). Processed rRNA sequences were mapped onto the corresponding reference trees using pplacer (52) with the default settings. The number of the mapped sequences to trees nodes was normalized to the total number of mapped sequences from the corresponding samples. Normalized abundances were visualized as circles mapped onto the reference trees such that the diameters of the circles were proportion to the taxonomic abundances.

Supplementary Results and Discussion

A molecular window into biogeochemistry

Several lines of evidence indicated that during the drift cells were experiencing iron-limitation and that this factor shaped community composition. WGCNA was used to examine functional clusters related to nutrient cycling (Figure S5). The expression of several low-iron response genes, including iron-starvation-induced proteins ISIP1, ISIP2A (phytotransferrin; (53), ISIP2B, and ISIP3 (54) in diatoms and haptophytes (module 5) indicated cellular iron stress. Phytotransferrin and “silicon transporter”

annotations clustered into the same module these iron-response genes (Figure S5; module 5; green) and were dominated by centric diatom expression. Module 5 peaks sharply at the end of the drift track when the measured silica:nitrate ratio, which was initially around 1, dropped most dramatically (Figure S1B). Low silica:nitrate ratios (in the range of 0.8 to 1.1) have been observed in association with iron limitation (55) and are thought to result from silica draw-down by iron-stressed diatoms (56). In our study, this ratio dropped by an order of magnitude along the drift track. While the main feature of module 5 is its sharp peak at the end of the drift track, there also appears to be some underlying diel periodicity in the signal (upregulated more during night hours). This convolution of expression patterns speaks to the difficulty of teasing apart various physical drivers of transcription in a dynamic natural context. In the future, sampling for a longer period of time could provide the statistical resolution to address these questions more conclusively.

Additional molecular evidence supported iron-limitation, such as high expression of iron complex outer membrane receptor proteins, which are associated with the uptake of siderophores (57), especially in the small fraction. Indeed, “iron complex outer membrane receptor protein” was the 34th most highly expressed annotation across both size classes (Supplementary Data 6). Furthermore, nitrate transporters and reductases were nearly undetectable across phytoplankton lineages whereas ammonium transporters were highly expressed, reflecting a reduced capacity for nitrate assimilation, which requires iron-rich heme cofactors (54). Finally, relative levels of ferredoxin and flavodoxin among photosynthetic organisms are often used as an indicator of iron stress (58,59). Iron-intensive ferredoxin proteins can be substituted by

flavodoxin, which performs the same role in photosynthesis but uses flavin mononucleotides in place of iron-sulfur clusters. Strikingly, in the large fraction, 98.12% of expression of these ORFs was attributed to flavodoxin (Pfam PF00258) over ferredoxin (Pfam PF00111) across the five major eukaryotic phytoplankton lineages (diatoms, chlorophytes, dinoflagellates, haptophytes, and pelagophytes; Figure S5). This ratio falls at the extreme edge of the distribution of previously observed cases (60), associated with the lowest iron concentrations. While direct trace-metal clean measurement of iron concentrations was not possible due to the nature of the robotic sampling, the gene sensors, historic oceanographic context (Figure S4), and nutrient proxies we present here establish a high likelihood of iron limitation.

Timing of diel ORF expression across taxonomic groups

Periodic ORFs were only detected in Proteobacteria known to possess proteorhodopsin or carry out anoxygenic photosynthesis. However, previous observations that benefitted from a longer time course were able to detect a greater diversity of periodic ORFs in heterotrophic bacterioplankton (22), indicating that these results only capture the strongest oscillating genes. We observed only a single periodic ORF in the photoheterotrophic bacteria, SAR11 and SAR116, both peaking around 12:30p.m. In SAR11, this ORF was in the isocitrate lyase family. This points to a daytime-shifted metabolism resulting from phototrophy; Glyoxylate shunt genes have been found to be expressed 300-fold more in light than darkness in the proteorhodopsin phototroph, *Dokdonia* (61), and were up during the day in photoheterotrophic bacterioplankton in a previous drift track (22). With the exception of a *Pseudomonas*

marR family transcriptional regulator peaking around 10a.m., all other periodic proteobacterial ORFs occurred in *Rhodobacter* species and were involved in nighttime chelatase, light-independent protochlorophyllide reductase, bacteriochlorophyll synthase, amine oxidases involved in carotenoid biosynthesis, diheme cytochrome c).

Viruses in the small size class

In the small fraction, bacteriophages corresponding to several of the abundant bacterial groups were enriched (e.g. *Pelagibacter* phage, $log_2FC=11$; *Roseobacter* phage, $log_2FC=7.4$; *Vibrio* phage, $log_2FC=5.2$; Supplementary Dataset 10), as was a virus best annotated as the uncultivated *Ostreococcus* OsV5 virus for which the host *Ostreococcus* clade is still unclear (15). However, despite the picoprasinophytes *Ostreococcus* ($log_2FC=2.25$) and *Bathycoccus* ($log_2FC=2.36$) being enriched in the small size class, the largest signal for viruses most similar to isolates known to infect them (i.e. OlV1, OtV1, and BpV1), came from the large fraction. This could indicate that the close proximity of cells in particle-associated microenvironments promotes infection, that infected cells more easily attach to particles (13), or that infected hosts are larger in size.

References

1. Ottesen EA, Young CR, Eppley JM, Ryan JP, Chavez FP, Scholin CA, et al. Pattern and synchrony of gene expression among sympatric marine microbial populations. Proc Natl Acad Sci. 2013 Feb 5;110(6):E488–97.

2. Scholin CA, Birch J, Jensen S, III RM, Massion E, Pargett D, et al. The quest to develop ecogenomic sensors: A 25-year history of the Environmental Sample Processor (ESP) as a case study. Oceanography. 2017;30(4):100–13.

3. Timothy Pennington J, Chavez FP. Seasonal fluctuations of temperature, salinity, nitrate, chlorophyll and primary production at station H3/M1 over 1989-1996 in Monterey Bay, California. Deep Res Part II Top Stud Oceanogr. 2000;47(5–6):947–73.

4. Lane DJ, Pace B, Olsen GJ, Stahl DA, Sogin ML, Pace NR. Rapid determination of 16S ribosomal RNA sequences for phylogenetic analyses. Proc Natl Acad Sci. 1985;82(20):6955–9.

5. Herlemann DPR, Labrenz M, Jürgens K, Bertilsson S, Waniek JJ, Andersson AF. Transitions in bacterial communities along the 2000 km salinity gradient of the Baltic Sea. ISME J. 2011;5(10):1571–9.

6. Stoeck T, Bass D, Nebel M, Christen R, Jones MDM, Breiner HW, et al. Multiple marker parallel tag environmental DNA sequencing reveals a highly complex eukaryotic community in marine anoxic water. Mol Ecol. 2010;19(SUPPL. 1):21–31.

7. Bertrand EM, McCrow JP, Moustafa A, Zheng H, McQuaid JB, Delmont TO, et al. Phytoplankton-bacterial interactions mediate micronutrient colimitation at the coastal Antarctic sea ice edge. Proc Natl Acad Sci U S A. 2015 Aug 11;112(32):9938–43.

8. Schmieder R, Lim YW, Edwards R. Identification and removal of ribosomal RNA sequences from metatranscriptomes. Bioinformatics. 2012 Feb 1;28(3):433–5.

9. Rho M, Tang H, Ye Y. FragGeneScan: predicting genes in short and error-prone reads. Nucleic Acids Res [Internet]. 2010 Nov 1 [cited 2016 Dec 1];38(20):e191–e191. Available from: https://academic.oup.com/nar/article-lookup/doi/10.1093/nar/gkq747

10. Protein BLAST: search protein databases using a protein query [Internet]. Available from: https://blast.ncbi.nlm.nih.gov/Blast.cgi?PAGE=Proteins

11. Altschul SF, Gish W, Miller W, Myers EW, Lipman DJ. Basic local alignment search tool. J Mol Biol [Internet]. 1990 Oct [cited 2017 Jul 17];215(3):403–10. Available from: http://linkinghub.elsevier.com/retrieve/pii/S0022283605803602

12. Sonnhammer E, Eddy SR, Birney E, Bateman A, Durbin R. Pfam: multiple sequence alignments and HMM-profiles of protein domains. Nucleic Acids Res. 1998 Jan 1;26(1):320–2.

13. Allen LZ, Allen EE, Badger JH, McCrow JP, Paulsen IT, Elbourne LD, et al. Influence of nutrients and currents on the genomic composition of microbes across an upwelling mosaic. ISME J. 2012;6(7):1403–14.

14. Dupont CL, Mccrow JP, Valas R, Moustafa A, Walworth N, Goodenough U, et al. Genomes and gene expression across light and productivity gradients in eastern subtropical Pacific microbial communities. ISME J. 2014;doi(10).

15. Zeigler Allen L, McCrow JP, Ininbergs K, Dupont CL, Badger JH, Hoffman JM, et al. The Baltic Sea virome: diversity and transcriptional activity of DNA and RNA viruses. mSystems. 2017;2(1):e00125-16.

16. Podell S, Gaasterland T. DarkHorse: A method for genome-wide prediction of horizontal gene transfer. Genome Biol. 2007;8(2).

17. Bender SJ, Moran DM, McIlvin MR, Zheng H, McCrow JP, Badger J, et al. Colony formation in *Phaeocystis antarctica*: Connecting molecular mechanisms with iron biogeochemistry. Biogeosciences. 2018;15(16):4923–42.
18. Bayat A, Gaëta B, Ignjatovic A, Parameswaran S. Improved VCF normalization for accurate VCF comparison. Bioinformatics. 2017 Mar 16;33(7):964–70.
19. Li H, Durbin R. Fast and accurate short read alignment with Burrows-Wheeler transform. Bioinformatics. 2009 Jul 15;25(14):1754–60.
20. Enright AJ, Van Dongen S, Ouzounis CA. An efficient algorithm for large-scale detection of protein families. Nucleic Acids Res. 2002;30(7):1575–84.
21. Ottesen EA, Young CR, Gifford SM, Eppley JM, Marin R, Schuster SC, et al. Multispecies diel transcriptional oscillations in open ocean heterotrophic bacterial assemblages. Science (80-). 2014;345(6193).
22. Aylward FO, Eppley JM, Smith JM, Chavez FP, Scholin CA, DeLong EF. Microbial community transcriptional networks are conserved in three domains at ocean basin scales. Proc Natl Acad Sci. 2015;112(17):5443–8.
23. R Development Core T e. a. m. R: a language and environment for statistical computing [Internet]. Vienna, Austria: R Foundation for Statistical Computing; 2011. Available from: http://www.r-project.org/
24. Benjamini Y, Hochberg Y. Controlling the false discovery rate: a practical and powerful approach to multiple testing. J R Stat Soc. 1995;57(1):289–300.
25. Langfelder P, Horvath S. WGCNA: An R package for weighted correlation network analysis. BMC Bioinformatics. 2008;9.
26. Zhang B, Horvath S. A General Framework for Weighted Gene Co-Expression

Network Analysis. Stat Appl Genet Mol Biol. 2005;4(1).
27. Csárdi G, Nepusz T. The igraph software package for complex network research. [cited 2017 Jul 26]; Available from: http://www.necsi.edu/events/iccs6/papers/c1602a3c126ba822d0bc4293371c.pdf
28. Robinson MD, McCarthy DJ, Smyth GK. edgeR: A Bioconductor package for differential expression analysis of digital gene expression data. Bioinformatics. 2009 Jan 1;26(1):139–40.
29. Jones P, Binns D, Chang HY, Fraser M, Li W, McAnulla C, et al. InterProScan 5: Genome-scale protein function classification. Bioinformatics. 2014;30(9):1236–40.
30. Glantz ST, Carpenter EJ, Melkonian M, Gardner KH, Boyden ES, Wong GK-S, et al. Functional and topological diversity of LOV domain photoreceptors. Proc Natl Acad Sci. 2016;113(11):E1442–51.
31. Edgar RC. MUSCLE: Multiple sequence alignment with high accuracy and high throughput. Nucleic Acids Res. 2004;32(5):1792–7.
32. Guindon S, Lethiec F, Duroux P, Gascuel O. PHYML Online - A web server for fast maximum likelihood-based phylogenetic inference. Nucleic Acids Res. 2005;33(SUPPL. 2).
33. Letunic I, Bork P. Interactive tree of life (iTOL) v3: an online tool for the display and annotation of phylogenetic and other trees. Nucleic Acids Res. 2016;44(W1):W242–5.
34. Agostinelli C, Lund U. R package “circular”: Circular Statistics [Internet]. 2017. Available from: https://r-forge.r-project.org/projects/circular/
35. Tasdan F, Yeniay O. Power study of circular anova test against nonparametric

alternatives. Hacettepe J Math Stat. 2014;43(1):97–115.

36. Wickham H. The split-apply-combine strategy for data analysis. J Stat Softw. 2011;40(1).

37. Wickham H, Francois R, Henry L, Müller K. dplyr: A grammar of data manipulation. 2017.

38. Wickham H. Reshaping data with the reshape package. 2006 [cited 2017 Jul 25]; Available from: http://had.co.nz/reshape

39. Wickham H. Ggplot2 : elegant graphics for data analysis [Internet]. Springer; 2009 [cited 2017 Jul 25]. 212 p. Available from: http://www.citeulike.org/group/18896/article/6995399

40. Grolemund G, Wickham H. Dates and times made easy with lubridate. JSS J Stat Softw [Internet]. 2011 [cited 2017 Jul 25];40(3). Available from: http://www.jstatsoft.org/

41. Kahle D, Wickham H. ggmap: Spatial visualization with ggplot2. R J [Internet]. 2013;5(1):144–61. Available from: http://journal.r-project.org/archive/2013-1/kahle-wickham.pdf

42. Auguie B. Miscellaneous functions for “grid” graphics [Internet]. 2016. p. 10. Available from: https://github.com/baptiste/gridextra

43. Edgar RC. Search and clustering orders of magnitude faster than BLAST. Bioinformatics. 2010;26(19):2460–1.

44. Mahé F, Rognes T, Quince C, de Vargas C, Dunthorn M. Swarm v2: highly-scalable and high-resolution amplicon clustering. PeerJ. 2015;3:e1420.

45. Pearson WR. Finding protein and nucleotide similarities with FASTA. Curr Protoc

Bioinforma. 2016;2016(March):3.9.1-3.9.25.

46. Quast C, Pruesse E, Yilmaz P, Gerken J, Schweer T, Yarza P, et al. The SILVA ribosomal RNA gene database project: Improved data processing and web-based tools. Nucleic Acids Res. 2013;41(D1):590–6.

47. De Vargas C, Audic S, Henry N, Decelle J, Mahé F, Logares R, et al. Eukaryotic plankton diversity in the sunlit ocean. Science (80-). 2015;348(6237):1261605.

48. Nawrocki EP, Eddy SR. Infernal 1.1: 100-fold faster RNA homology searches. Bioinformatics. 2013 Nov;29(22):2933–5.

49. Katoh K, Standley DM. MAFFT multiple sequence alignment software version 7: improvements in performance and usability. Mol Biol Evol. 2013 Apr;30(4):772–80.

50. Waterhouse AM, Procter JB, Martin DMA, Clamp M, Barton GJ. Jalview Version 2-A multiple sequence alignment editor and analysis workbench. Bioinformatics. 2009 May;25(9):1189–91.

51. Price MN, Dehal PS, Arkin AP. FastTree 2 - Approximately maximum-likelihood trees for large alignments. PLoS One. 2010 Mar;5(3):e9490.

52. Matsen FA, Kodner RB, Armbrust EV. pplacer: linear time maximum-likelihood and Bayesian phylogenetic placement of sequences onto a fixed reference tree. BMC Bioinformatics. 2010 Oct;11:538.

53. McQuaid JB, Kustka AB, Oborník M, Horák A, McCrow JP, Karas BJ, et al. Carbonate-sensitive phytotransferrin controls high-affinity iron uptake in diatoms. Nature. 2018;555(7697):534–7.

54. Allen AE, LaRoche J, Maheswari U, Lommer M, Schauer N, Lopez PJ, et al.

Whole-cell response of the pennate diatom Phaeodactylum tricornutum to iron starvation. Proc Natl Acad Sci. 2008;105(30):10438–43.

55. Hutchins DA, Bruland KW. Iron-limited diatom growth and Si:N uptake ratios in a coastal upwelling regime. Nature. 1998 Jun 11;393(6685):561–4.

56. Brzezinski MA, Krause JW, Bundy RM, Barbeau KA, Franks P, Goericke R, et al. Enhanced silica ballasting from iron stress sustains carbon export in a frontal zone within the California Current. J Geophys Res C Ocean. 2015;120(7):4654–69.

57. Tang K, Jiao N, Liu K, Zhang Y, Li S. Distribution and functions of tonb-dependent transporters in marine bacteria and environments: Implications for dissolved organic matter utilization. PLoS One. 2012;7(7).

58. McKay RML, La Roche J, Yakunin AF, Durnford DG, Geider RJ. Accumulation of ferredoxin and flavodoxin in a marine diatom in response to Fe. J Phycol. 1999;35(3):510–9.

59. Erdner DL, Anderson DM. Ferredoxin and flavodoxin as biochemical indicators of iron limitation during open-ocean iron enrichment. Limnol Oceanogr. 1999;44(7):1609–15.

60. Carradec Q, Pelletier E, Da Silva C, Alberti A, Seeleuthner Y, Blanc-Mathieu R, et al. A global ocean atlas of eukaryotic genes. Nat Commun. 2018;9(1).

61. Palovaara J, Akram N, Baltar F, Bunse C, Forsberg J, Pedros-Alio C, et al. Stimulation of growth by proteorhodopsin phototrophy involves regulation of central metabolic pathways in marine planktonic bacteria. Proc Natl Acad Sci. 2014;111(35):E3650–8.
