# Supplementary material for: Diel transcriptional response of a California Current plankton microbiome to light, low iron, and enduring viral infection
Source: ISME J. 2019 Jul 18;13(11):2817–33. doi: 10.1038/s41396-019-0472-2 (PMC6794264; doi:10.1038/s41396-019-0472-2)
Supplement: Supplementary file 2 — S2: Supplemental figures [file 41396_2019_472_MOESM2_ESM.pdf]

Supplementary File 2:

Supplementary Figures

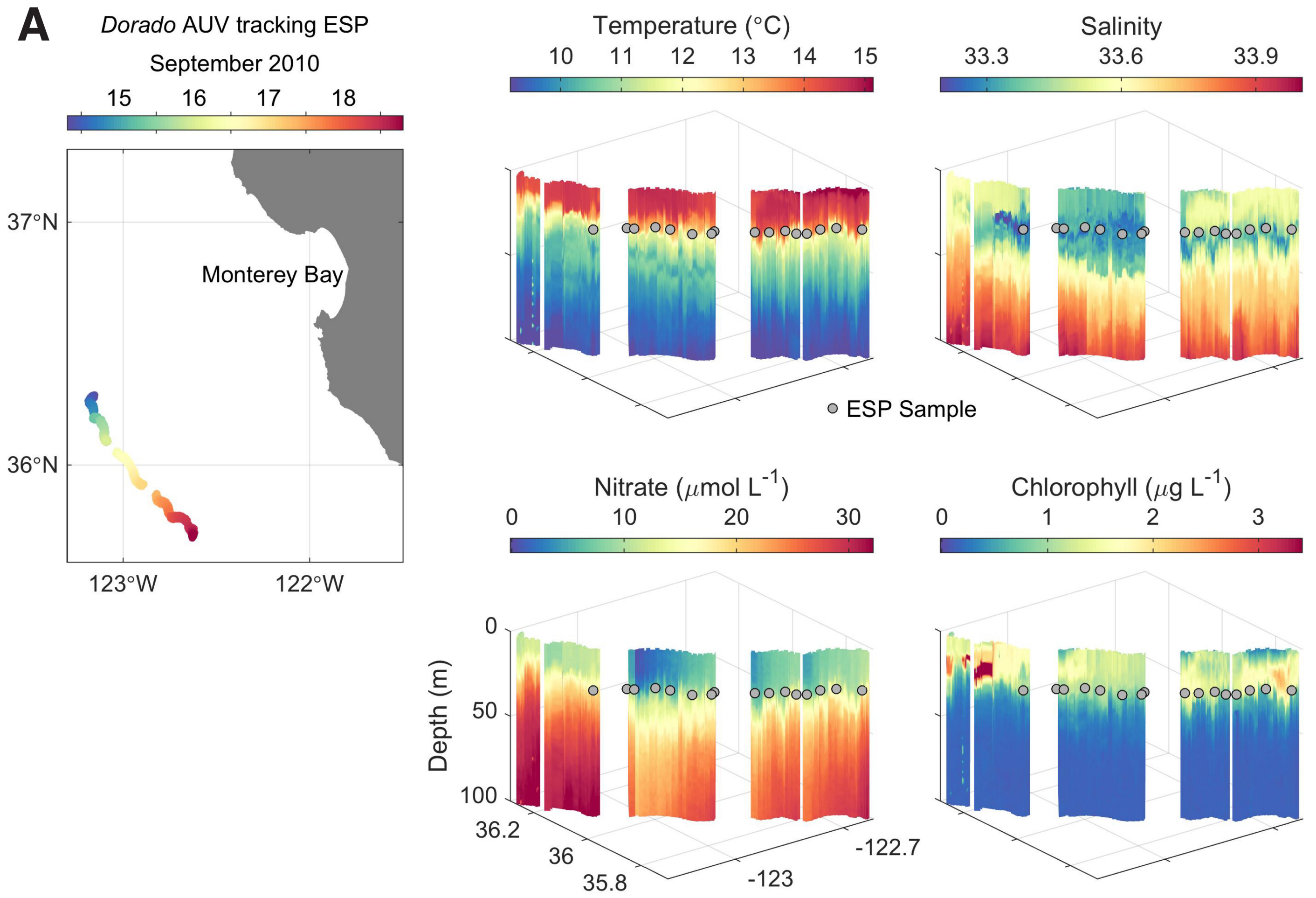

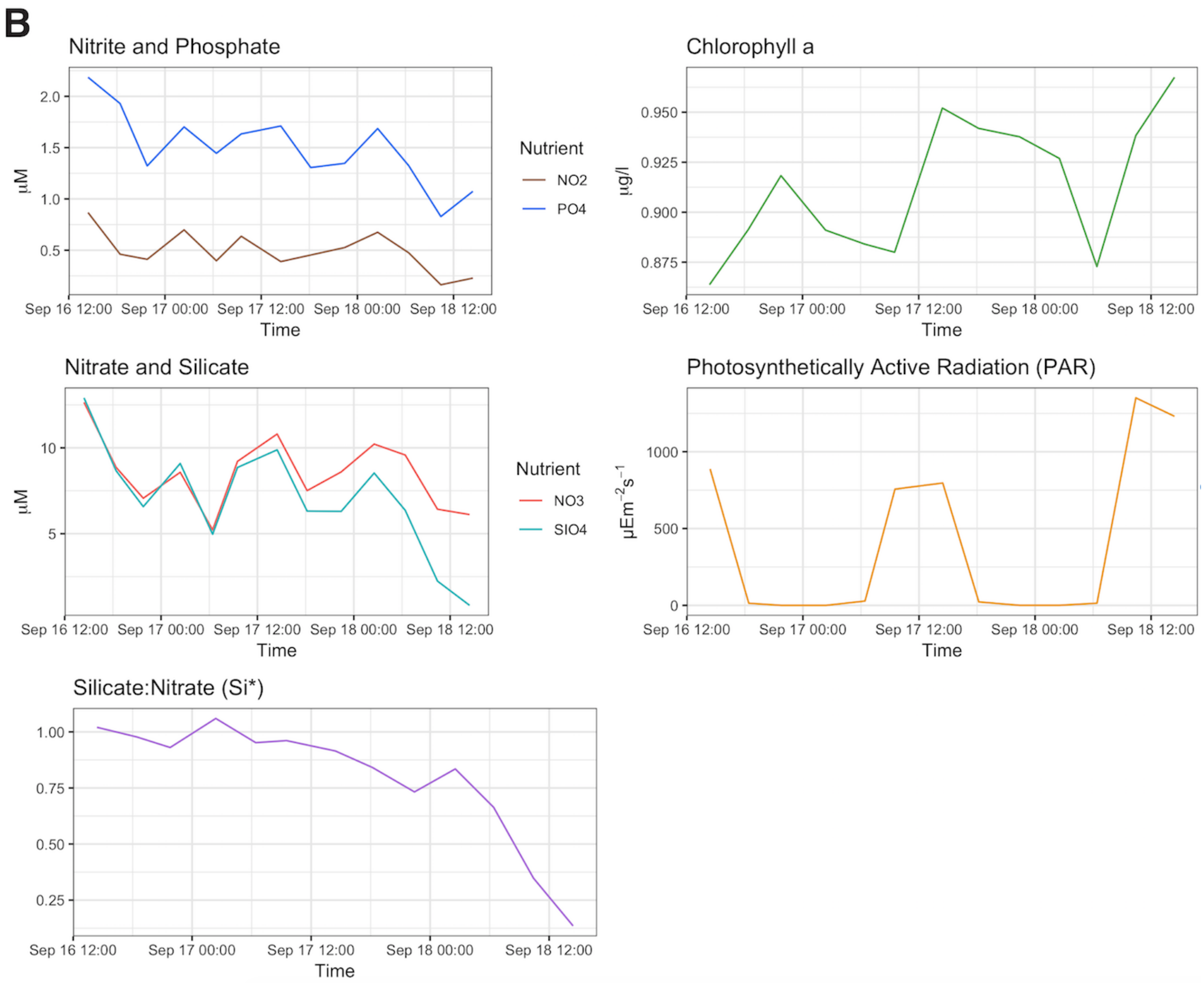

Figure S1 Biogeographical context of the drift track. **(A)** *In situ* oceanographic conditions of the drift track as observed by the *Dorado* AUV. Grey dots represent approximate locations of ESP samples. **(B)** Nutrients, chlorophyll, and light availability along the drift track at the depth of the ESP drift (~23m).

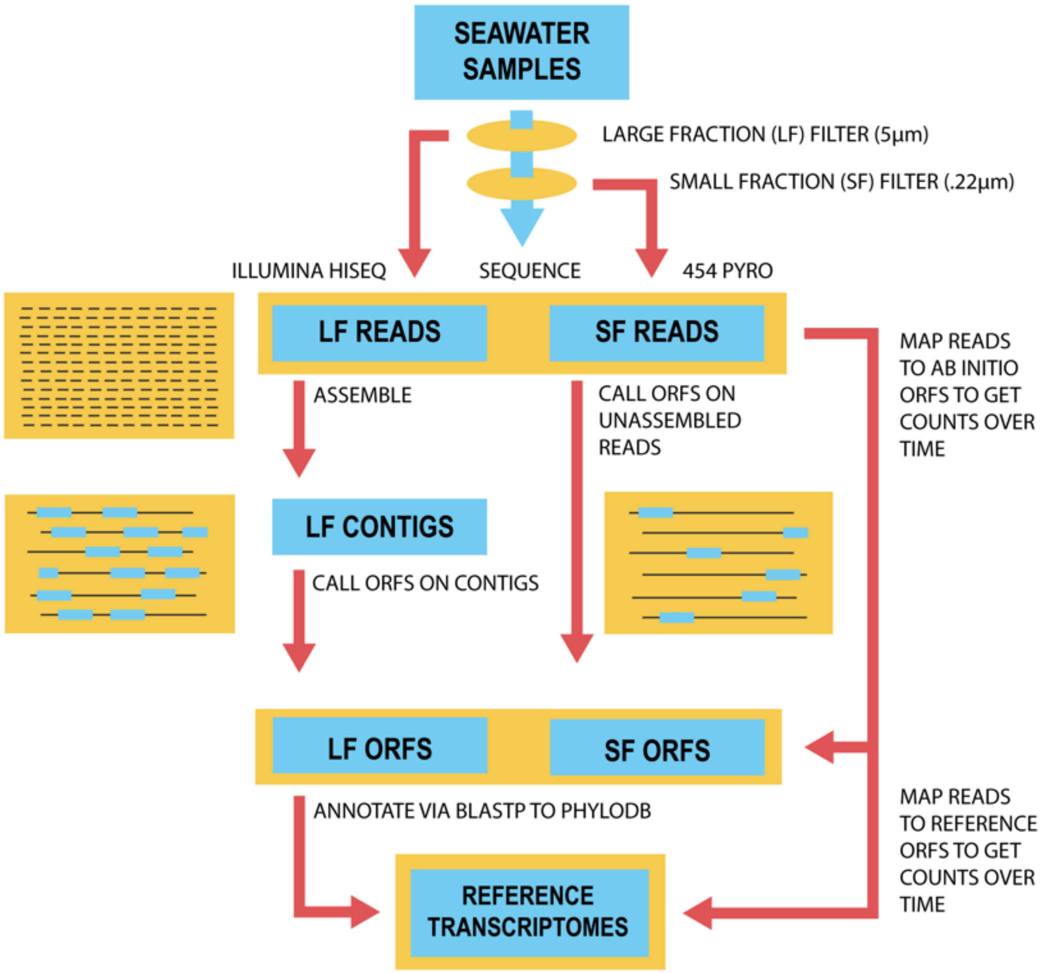

Figure S2 Bioinformatic pipeline. Seawater was collected onto 5µm and 0.22 µm filters, separating biomass into a large and small fraction, respectively. Large fraction (LF) reads were sequenced on the Illumina HiSeq platform, whereas small fraction (SF) reads had been previously sequenced by Ottesen *et al.* in 2012 on a GS FLX Titanium system (1). *Ab initio* ORF predictions were called on assembled large fraction contigs and directly on small fraction ORFs due to the longer read length, lower coverage nature of 454 sequencing. This less-restrictive amino acid space approach allowed us to map 7x more reads than traditional nucleotide space mapping to known references. Still, despite mapping 107 million reads, 158 million reads could not be mapped to *ab initio* ORFs, and those that did only averaged 67.2% identity to their best BLAST hit. Transcriptomes of reference organisms were chosen based on similarity and abundance of closely related species. Large and small fraction reads were mapped to reference transcriptomes using nucleotide Burrows Wheeler Aligner (BWA; (2). Reference transcriptomes were hierarchically clustered together with large and small fraction ORFs to gene ortholog groups. The resultant clusters, reference transcriptomes, and de-novo ORFs from both fractions were annotated taxonomically and functionally and used for downstream analyses, including pattern recognition algorithms such as Harmonic Regression Analysis (HRA) and Weighted Gene Network Correlation Analysis (WGCNA).

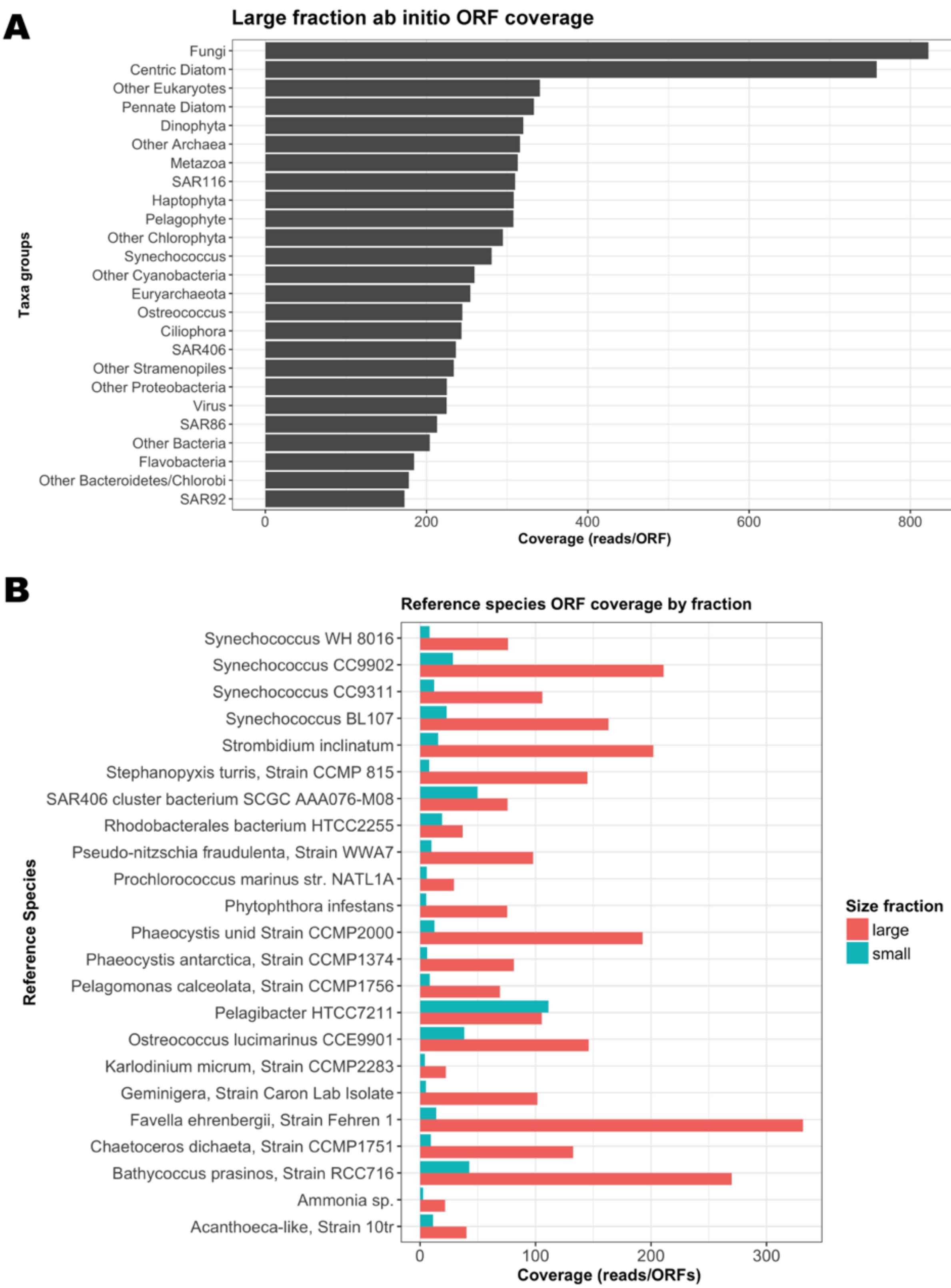

Figure S3 Depth of coverage of **(A)** large fraction *ab initio* ORFs by taxa group and **(B)** large and small fraction nucleotide references.

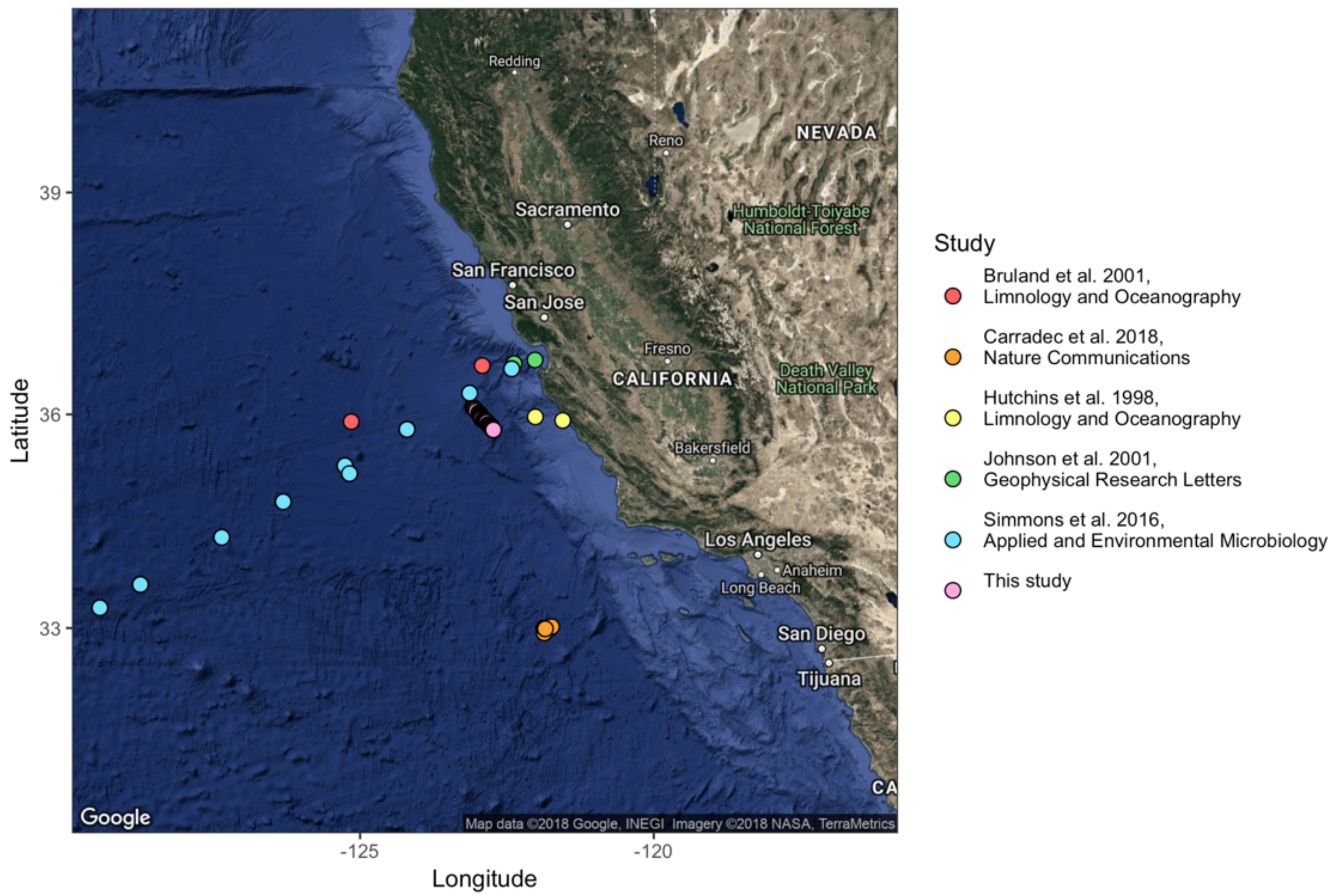

Figure S4 Location of drift track (pink) relative to sites with documented iron limitation (Supplementary Data 12E).
Red: July, 1995, measured total Fe < 0.05 nM, Fe-enrichment experiments confirm Fe limitation (3)
Orange: modeled total Fe < .04 umol/m3 based on global oceanographic data (4)
Yellow: June 1996/ June 1997, measured DPSCSV reactive Fe ≤ 0.1 nM, total Fe ≤ 0.1 nM, Fe-enrichment experiments confirm moderate to severe Fe limitation (5)
Green: Monterey Bay moorings M1 and M2, seasonal Fe limitation documented (e.g. June 1999/August 1999, measured total Fe < 1 nM) (6)
Blue: September-October 2009, total Fe < 1nM for at least 1 depth at given coordinates (7)

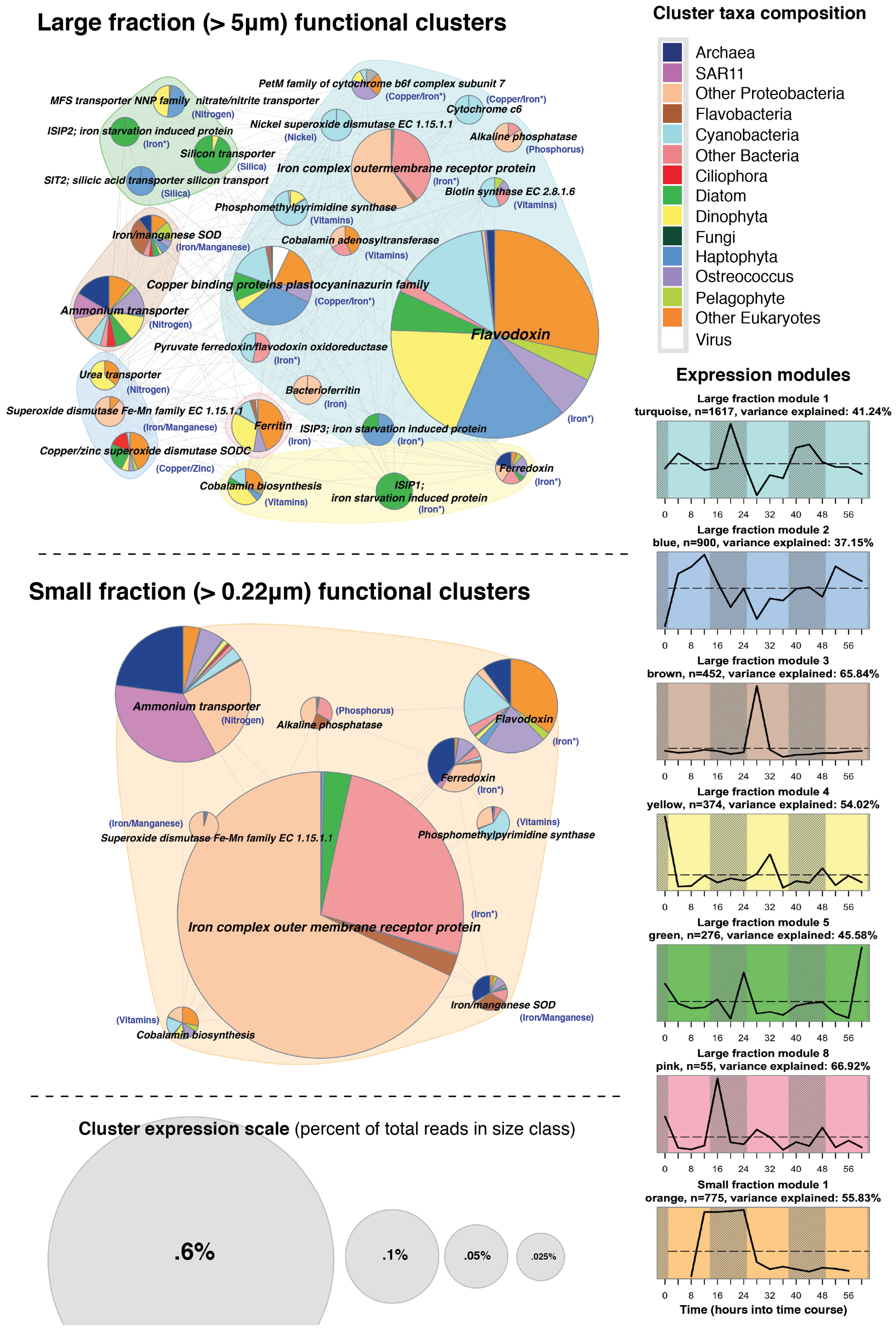

Large fraction (> 5µm) functional clusters
PetM family of cytochrome b6f complex subunit 7
(Copper/Iron*)
MFS transporter NNP family nitrate/nitrite transporter
(Nitrogen)
Cytochrome c6
(Copper/Iron*)
Nickel superoxide dismutase EC 1.15.1.1
(Nickel)
ISIP2; iron starvation induced protein
(Iron*)
Alkaline phosphatase
(Phosphorus)
Silicon transporter
(Silica)
Iron complex outermembrane receptor protein
(Iron*)
SIT2; silicic acid transporter silicon transport
(Silica)
Biotin synthase EC 2.8.1.6
(Vitamins)
Phosphomethylpyrimidine synthase
(Vitamins)
Iron/manganese SOD
(Iron/Manganese)
Cobalamin adenosyltransferase
(Vitamins)
Copper binding proteins plastocyaninazurin family
(Copper/Iron*)
Ammonium transporter
(Nitrogen)
Flavodoxin
Pyruvate ferredoxin/flavodoxin oxidoreductase
(Iron*)
Urea transporter
(Nitrogen)
Bacterioferritin
(Iron)
(Iron*)
Superoxide dismutase Fe-Mn family EC 1.15.1.1
(Iron/Manganese)
Ferritin
(Iron)
ISIP3; iron starvation induced protein
(Iron*)
Copper/zinc superoxide dismutase SODC
(Copper/Zinc)
Ferredoxin
(Iron*)
Cobalamin biosynthesis
(Vitamins)
ISIP1;
iron starvation induced protein
(Iron*)
Small fraction (> 0.22µm) functional clusters
Ammonium transporter
(Nitrogen)
(Phosphorus)
Alkaline phosphatase
Flavodoxin
(Iron*)
Ferredoxin
(Iron*)
(Iron/Manganese)
Superoxide dismutase Fe-Mn family EC 1.15.1.1
(Vitamins)
Phosphomethylpyrimidine synthase
(Iron*)
Iron complex outer membrane receptor protein
Iron/manganese SOD
(Iron/Manganese)
(Vitamins)
Cobalamin biosynthesis
Cluster expression scale (percent of total reads in size class)
.6%
.1%
.05%
.025%
Cluster taxa composition
Archaea
SAR11
Other Proteobacteria
Flavobacteria
Cyanobacteria
Other Bacteria
Ciliophora
Diatom
Dinophyta
Fungi
Haptophyta
Ostreococcus
Pelagophyte
Other Eukaryotes
Virus
Expression modules
Large fraction module 1
turquoise, n=1617, variance explained: 41.24%
Large fraction module 2
blue, n=900, variance explained: 37.15%
Large fraction module 3
brown, n=452, variance explained: 65.84%
Large fraction module 4
yellow, n=374, variance explained: 54.02%
Large fraction module 5
green, n=276, variance explained: 45.58%
Large fraction module 8
pink, n=55, variance explained: 66.92%
Small fraction module 1
orange, n=775, variance explained: 55.83%
0 8 16 24 32 40 48 56
Time (hours into time course)

Figure S5 Expression of major nutrient cycling genes across size classes. Pies represent annotated functional clusters of *ab initio* ORFs and are colored by relative taxonomic contribution. The biogeochemical pathway each cluster participates in is noted in blue; asterisks denote ORFs previously observed to be transcriptionally sensitive to iron limitation. Clusters are grouped by modules of similar expression as given by WGCNA.

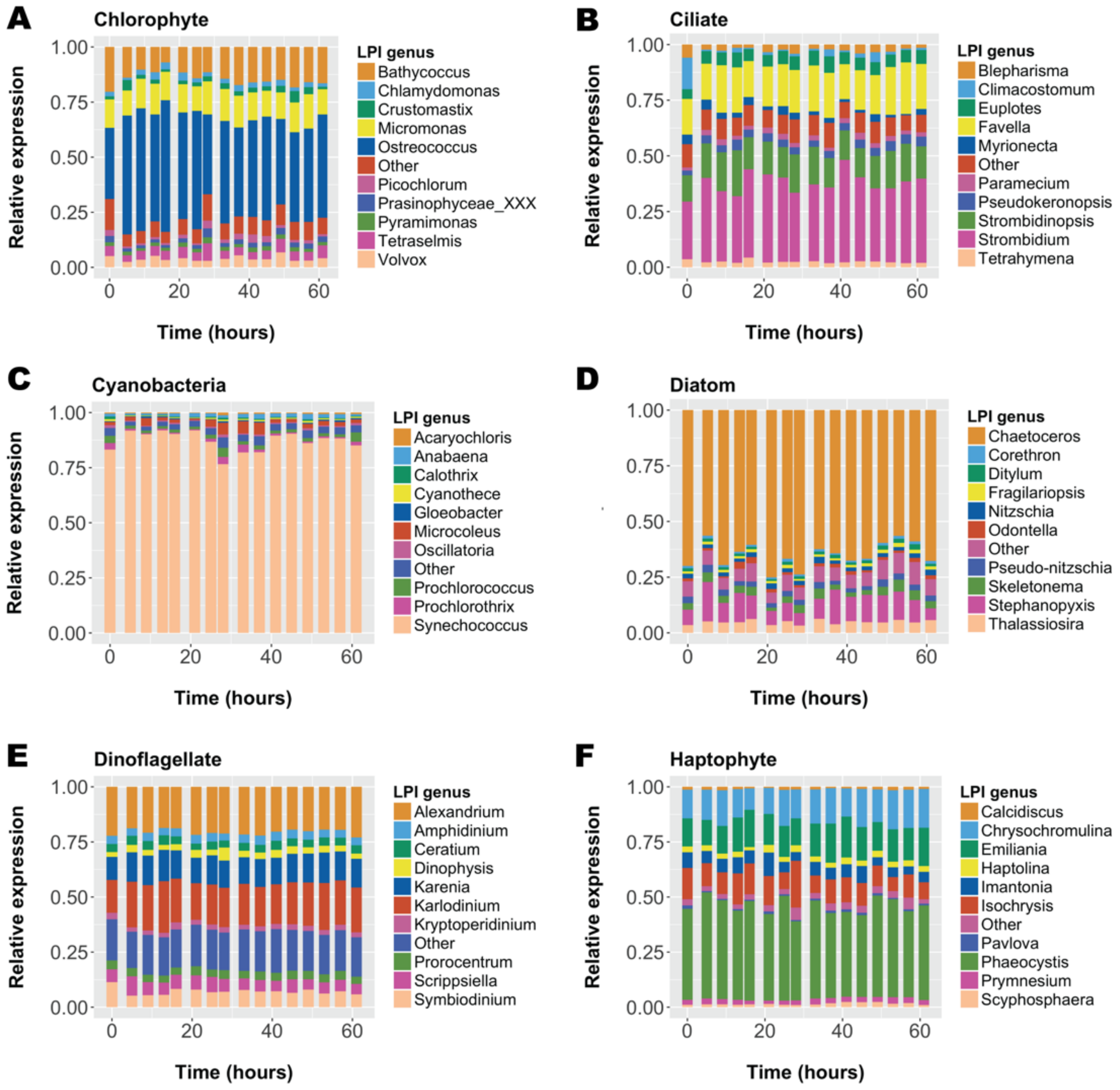

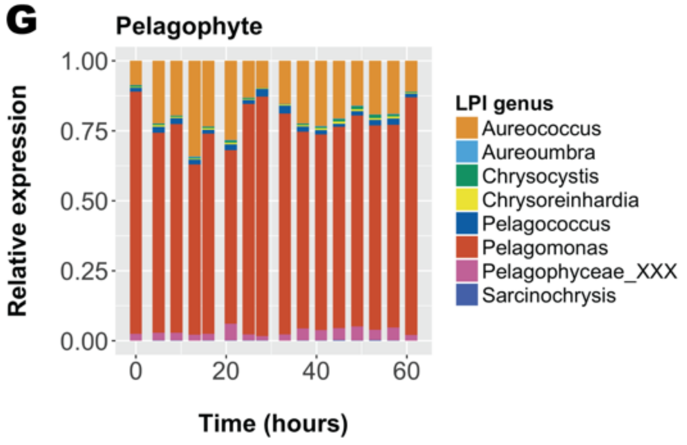

Figure S6 Genus level total community mRNA expression of major phytoplankton taxa (chlorophytes **(A)**, ciliates **(B)**, cyanobacteria **(C)**, diatoms **(D)**, dinoflagellates **(E)**, haptophytes **(F)**, and pelagophytes **(G)**) in the large size class. Expression is normalized by library (time point). Genus of each ORF is determined by best LPI hit.

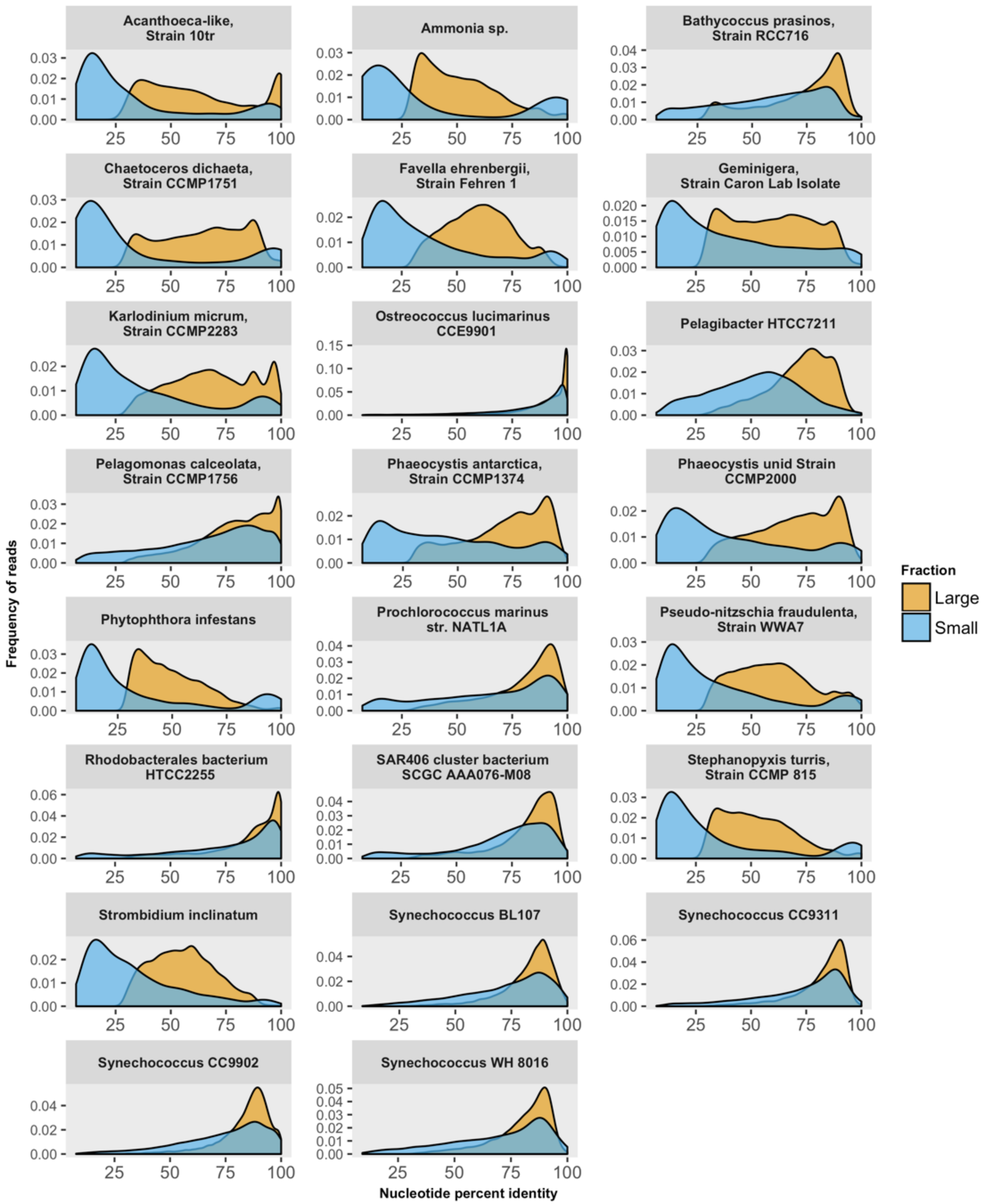

Figure S7 Average nucleotide percent identity of large fraction reads mapping to reference transcriptomes in the large (orange) and small (blue) size classes.

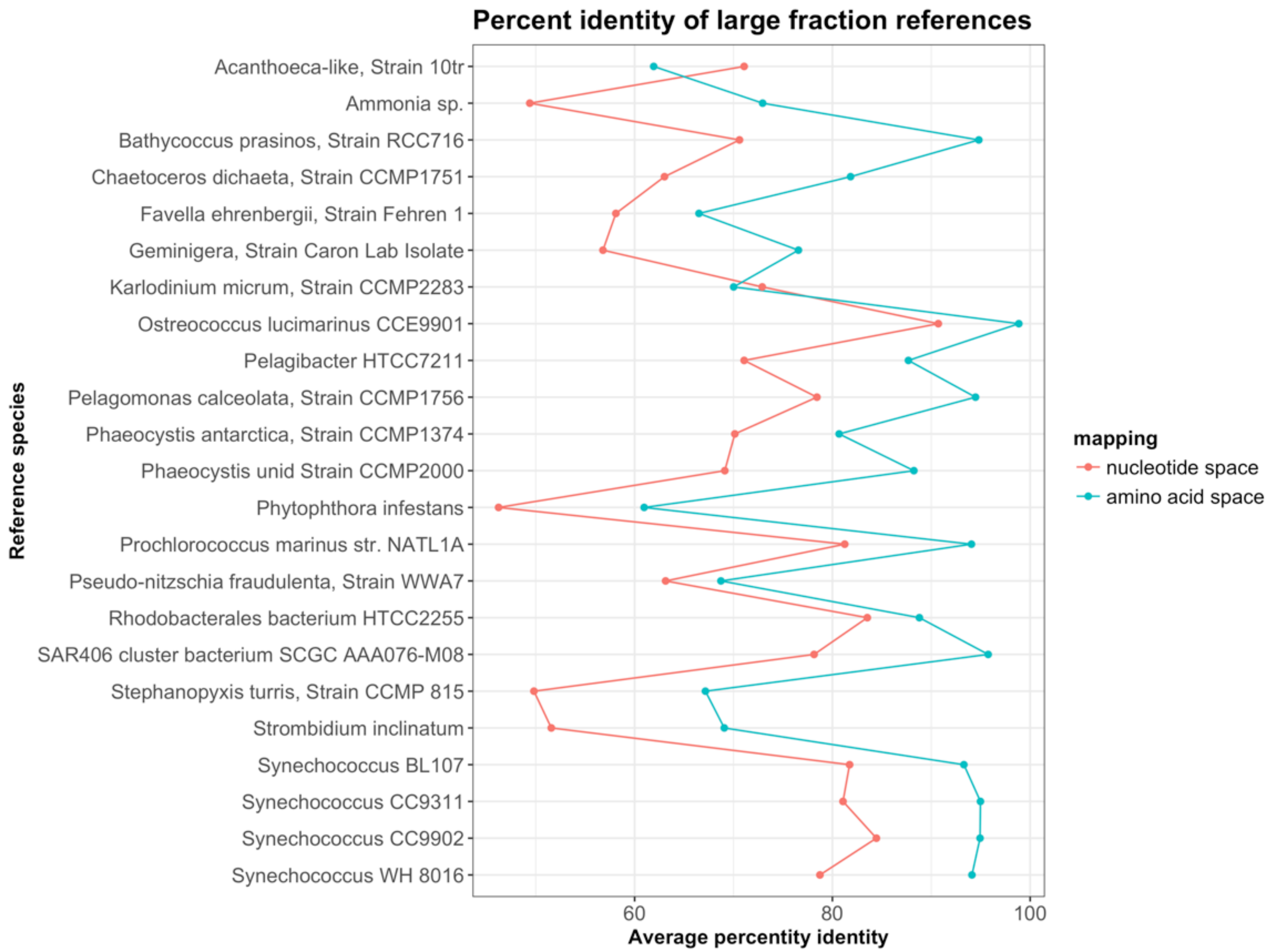

Figure S8 Comparison of average percent identity of reads mapping to reference transcriptomes in nucleotide space (red) and reads mapping to *ab initio* ORFs in amino acid space (blue).

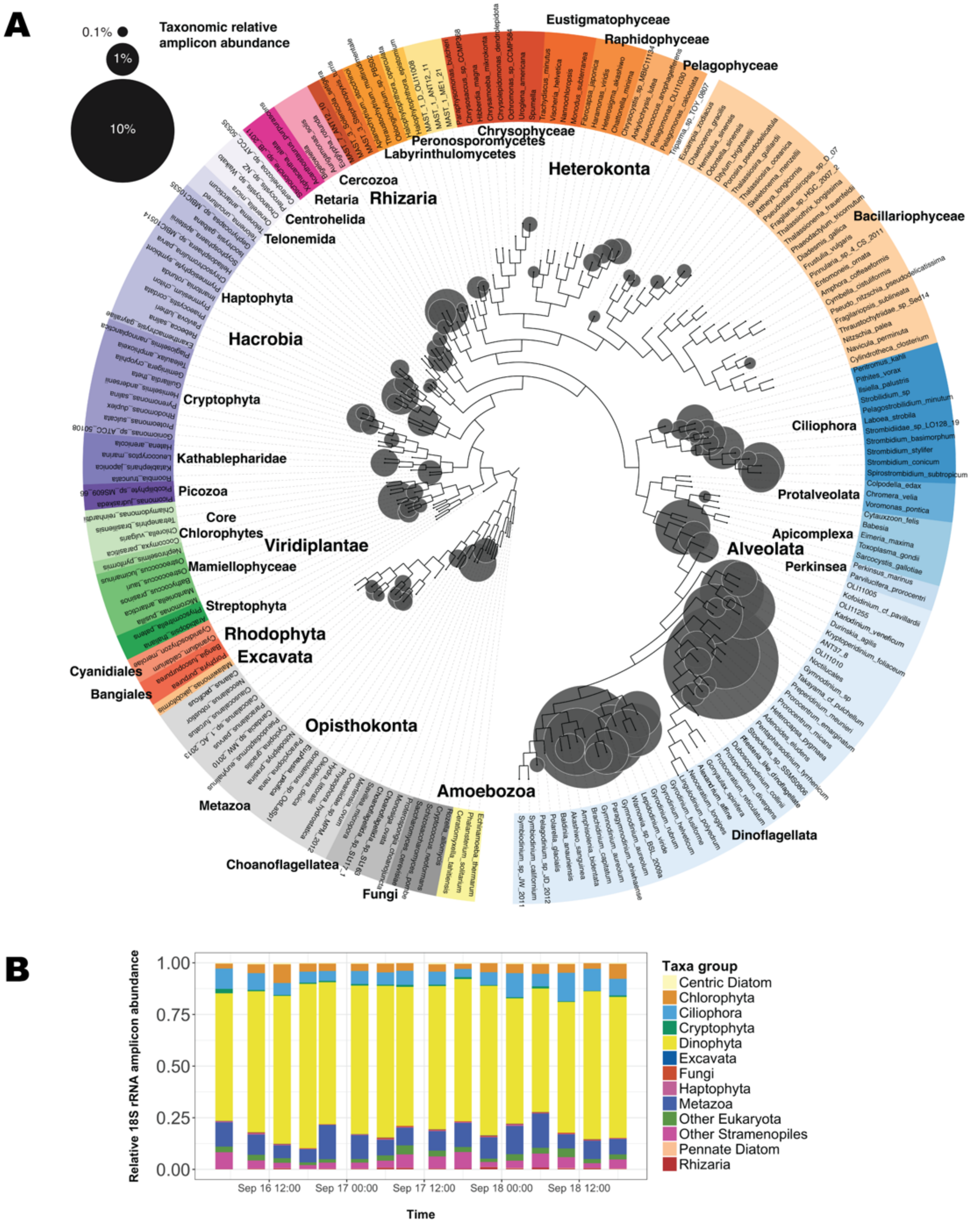

Figure S9 (A) Phylogenetic tree showing distribution of active large fraction eukaryotes using 18S rRNA amplicons (Supplementary Data 3). Circles representing relative amplicon abundance are superimposed over a reference phylogeny which is colored by taxonomy. Proximity of circles to the

tips of branches represents closeness to references. **(B)** Relative 18S rRNA amplicon abundance over time.

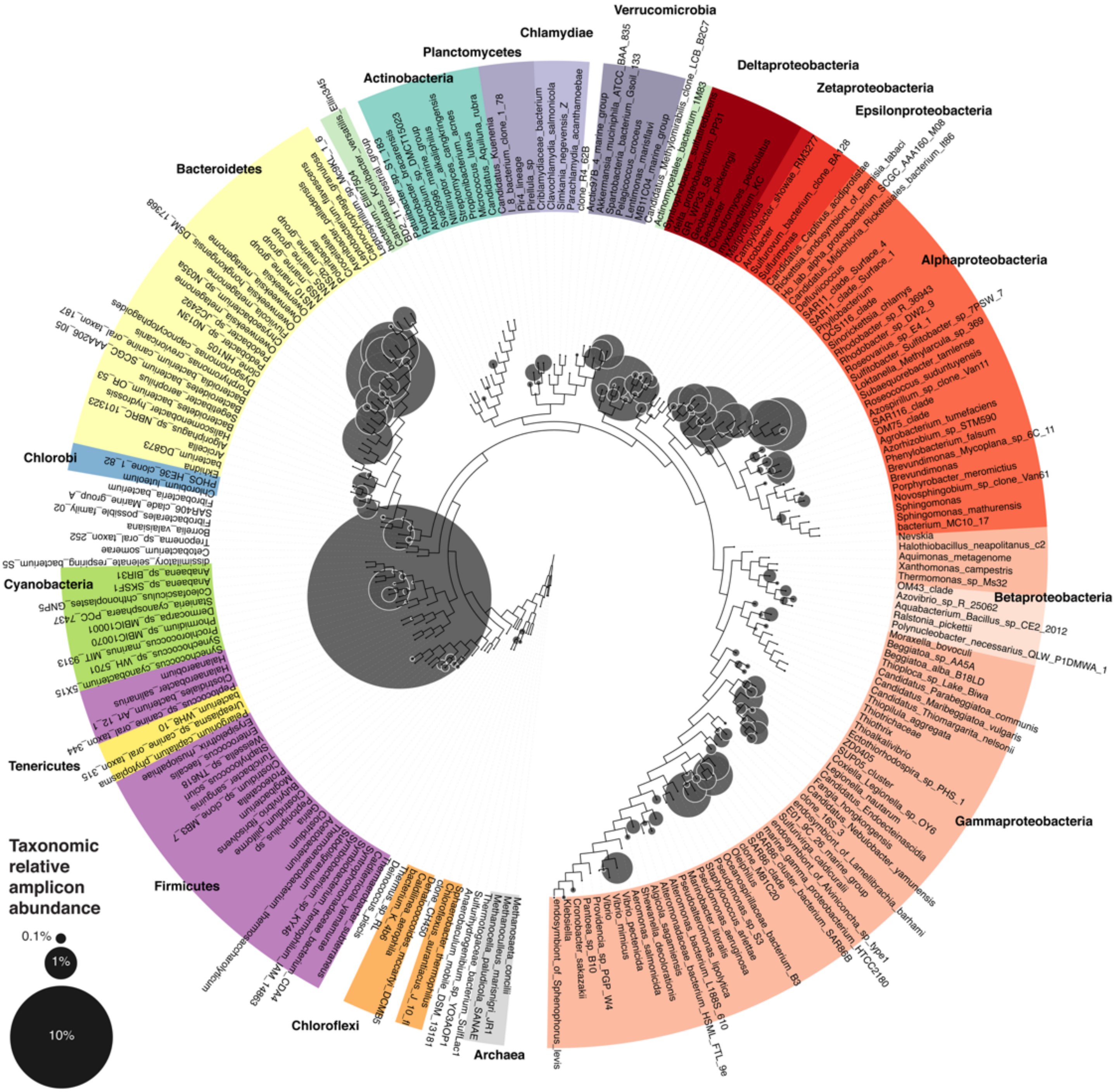

Figure S10 Phylogenetic tree showing distribution of active large fraction bacterial taxa using 16S rRNA amplicons (Supplementary Data 4). Circles representing relative amplicon abundance are superimposed over a reference phylogeny which is colored by taxonomy. Proximity of circles to the tips of branches represents closeness to references.

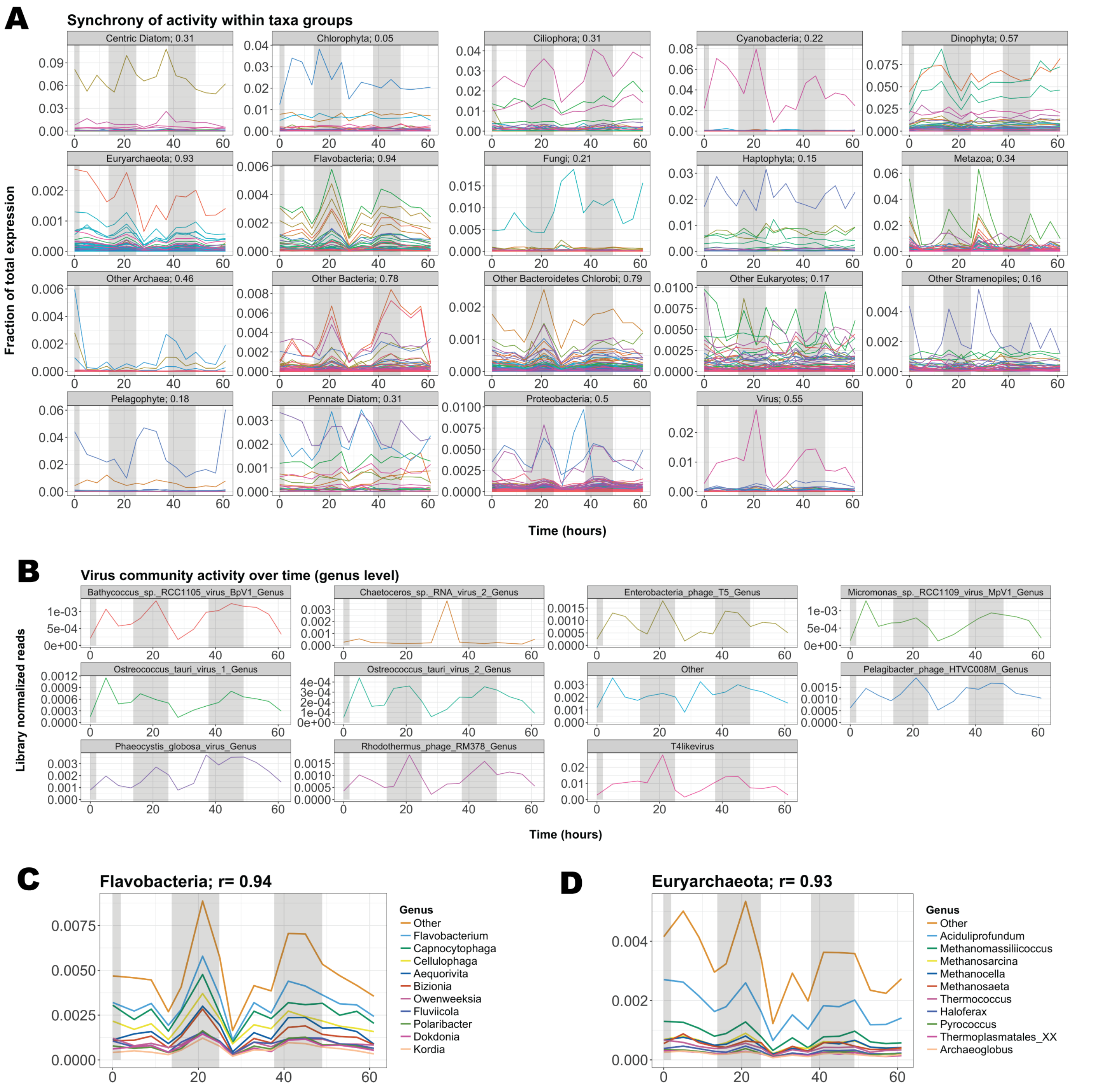

Figure S11 Synchronization of total activity among related organisms in the large fraction as viewed using *ab initio* ORFs. **(A)** Library (time point) normalized expression of *ab initio* ORFs binned by LPI-based taxonomic group. Numbers in headers denote strength of correlation between ORFs in a shared taxa group (Pearson's r). **(B)** Library normalized expression of top 10 large fraction virus genera. **(C)** and **(D)** show genus-level contributions of highly synchronous *Flavobacteria* and *Euryarchaeota* groups, respectively.

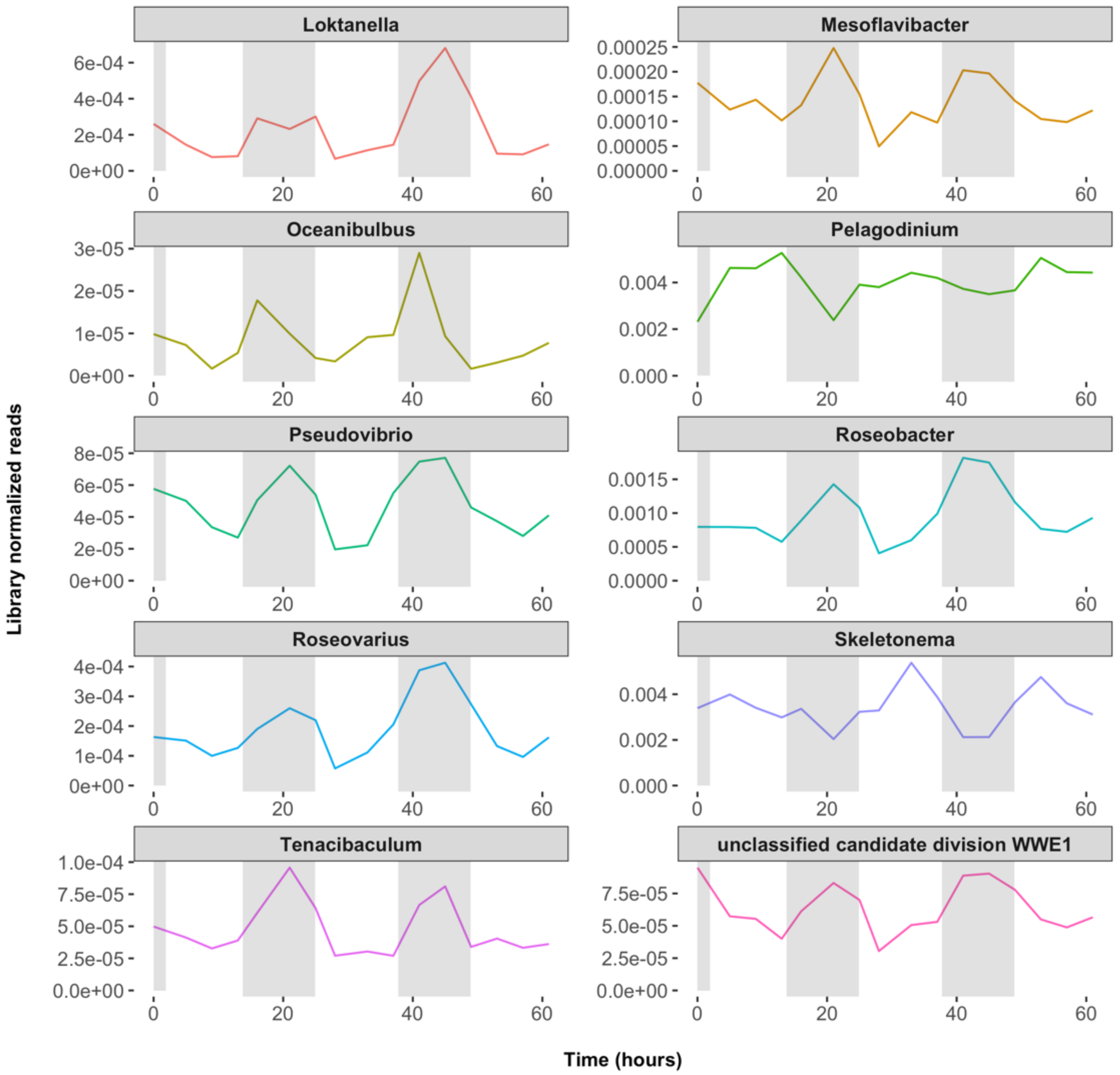

Figure S12 Total library (time point) normalized activity of large fraction genera that exhibit significant 24-h periodicity (HRA; FDR p ≤ 0.1). Two photosynthetic eukaryotes, *Pelagodinium*, a photosynthetic dinoflagellate symbiotic with foraminifera (8), and the centric diatom *Skeletonema*, had peak activity during the day. The remaining genera were non-photosynthetic bacteria with aggregate gene expression peaking at night: *Loktanella*, *Mesoflavibacter*, *Oceanibulbus*, *Pseudovibrio*, *Roseobacter*, *Roseovarius*, *Tenacibaculum*, and *Unclassified candidate division WWE1*. Several are known phytoplankton associates (e.g. *Loktanella spp.* (9,10)) and early particle colonizers (11) not previously known to operate on a diel cycle.

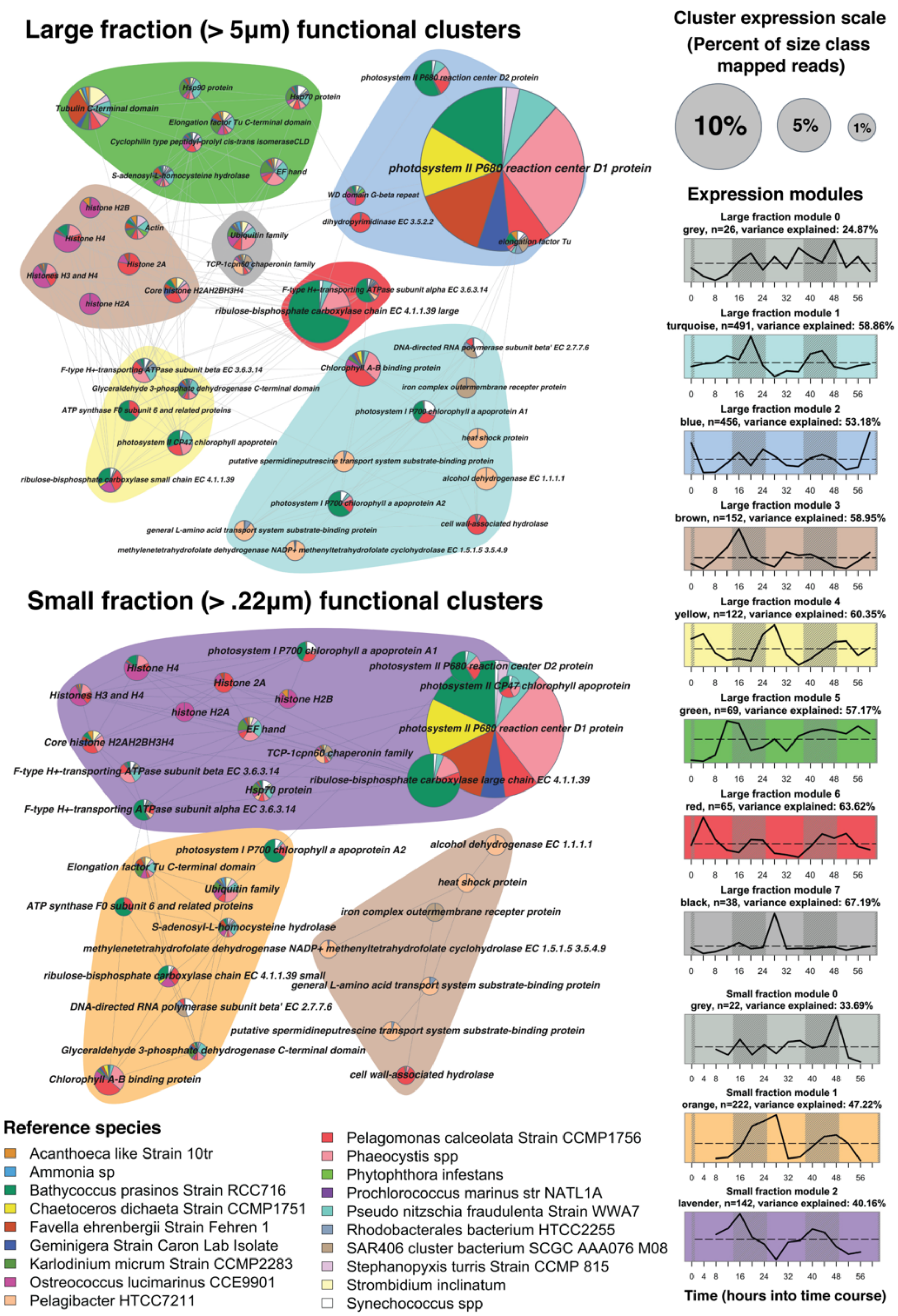

Large fraction (> 5μm) functional clusters
Tubulin C-terminal domain
Hsp90 protein
Hsp70 protein
Elongation factor Tu C-terminal domain
Cyclophilin type peptidyl-prolyl cis-trans isomeraseCLD
S-adenosyl-L-homocysteine hydrolase
EF hand
photosystem II P680 reaction center D2 protein
photosystem II P680 reaction center D1 protein
WD domain G-beta repeat
dihydropyrimidinase EC 3.5.2.2
elongation factor Tu
histone H2B
Actin
Histone H4
Ubiquitin family
Histone 2A
TCP-1cpn60 chaperonin family
Histones H3 and H4
Core histone H2AH2BH3H4
F-type H+-transporting ATPase subunit alpha EC 3.6.3.14
histone H2A
ribulose-bisphosphate carboxylase chain EC 4.1.1.39 large
DNA-directed RNA polymerase subunit beta' EC 2.7.7.6
F-type H+-transporting ATPase subunit beta EC 3.6.3.14
Chlorophyll A-B binding protein
Glyceraldehyde 3-phosphate dehydrogenase C-terminal domain
iron complex outermembrane recepter protein
ATP synthase F0 subunit 6 and related proteins
photosystem I P700 chlorophyll a apoprotein A1
heat shock protein
photosystem II CP42 chlorophyll apoprotein
putative spermidineputrescine transport system substrate-binding protein
alcohol dehydrogenase EC 1.1.1.1
ribulose-bisphosphate carboxylase small chain EC 4.1.1.39
photosystem I P700 chlorophyll a apoprotein A2
cell wall-associated hydrolase
general L-amino acid transport system substrate-binding protein
methylenetetrahydrofolate dehydrogenase NADP+ methenyltetrahydrofolate cyclohydrolase EC 1.5.1.5 3.5.4.9
Small fraction (> .22μm) functional clusters
photosystem I P700 chlorophyll a apoprotein A1
Histone H4
photosystem II P680 reaction center D2 protein
Histone 2A
Histones H3 and H4
histone H2B
photosystem II CP47 chlorophyll apoprotein
histone H2A
EF hand
photosystem II P680 reaction center D1 protein
Core histone H2AH2BH3H4
TCP-1cpn60 chaperonin family
F-type H+-transporting ATPase subunit beta EC 3.6.3.14
ribulose-bisphosphate carboxylase large chain EC 4.1.1.39
Hsp70 protein
F-type H+-transporting ATPase subunit alpha EC 3.6.3.14
photosystem I P700 chlorophyll a apoprotein A2
alcohol dehydrogenase EC 1.1.1.1
Elongation factor Tu C-terminal domain
heat shock protein
Ubiquitin family
ATP synthase F0 subunit 6 and related proteins
iron complex outermembrane recepter protein
S-adenosyl-L-homocysteine hydrolase
methylenetetrahydrofolate dehydrogenase NADP+ methenyltetrahydrofolate cyclohydrolase EC 1.5.1.5 3.5.4.9
ribulose-bisphosphate carboxylase chain EC 4.1.1.39 small
general L-amino acid transport system substrate-binding protein
DNA-directed RNA polymerase subunit beta' EC 2.7.7.6
putative spermidineputrescine transport system substrate-binding protein
Glyceraldehyde 3-phosphate dehydrogenase C-terminal domain
cell wall-associated hydrolase
Chlorophyll A-B binding protein
Reference species
Acanthoeca like Strain 10tr
Ammonia sp
Bathycoccus prasinos Strain RCC716
Chaetoceros dichaeta Strain CCMP1751
Favella ehrenbergii Strain Fehren 1
Geminigera Strain Caron Lab Isolate
Karlodinium micrum Strain CCMP2283
Ostreococcus lucimarinus CCE9901
Pelagibacter HTCC7211
Pelagomonas calceolata Strain CCMP1756
Phaeocystis spp
Phytophthora infestans
Prochlorococcus marinus str NATL1A
Pseudo nitzschia fraudulenta Strain WWA7
Rhodobacterales bacterium HTCC2255
SAR406 cluster bacterium SCGC AAA076 M08
Stephanopyxis turris Strain CCMP 815
Strombidium inclinatum
Synechococcus spp
Cluster expression scale
(Percent of size class mapped reads)
10%
5%
1%
Expression modules
Large fraction module 0
grey, n=26, variance explained: 24.87%
Large fraction module 1
turquoise, n=491, variance explained: 58.86%
Large fraction module 2
blue, n=456, variance explained: 53.18%
Large fraction module 3
brown, n=152, variance explained: 58.95%
Large fraction module 4
yellow, n=122, variance explained: 60.35%
Large fraction module 5
green, n=69, variance explained: 57.17%
Large fraction module 6
red, n=65, variance explained: 63.62%
Large fraction module 7
black, n=38, variance explained: 67.19%
Small fraction module 0
grey, n=22, variance explained: 33.69%
Small fraction module 1
orange, n=222, variance explained: 47.22%
Small fraction module 2
lavender, n=142, variance explained: 40.16%
0 8 16 24 32 40 48 56
0 4 8 16 24 32 40 48 56
Time (hours into time course)

Figure S13 A comparison of functional diversity across fractions by mapping reads to transcriptomes of cultured representatives. Pies represent most abundant functional clusters of reference ORFs. Pies are colored by relative taxonomic contribution and grouped by modules of similar expression as given by WGCNA. Note that reads mapping to *Favella ehrengbergii* Strain Fehren 1 (e.g. those involved in photosynthesis) may be hitting remnants of its photosynthetic food source.

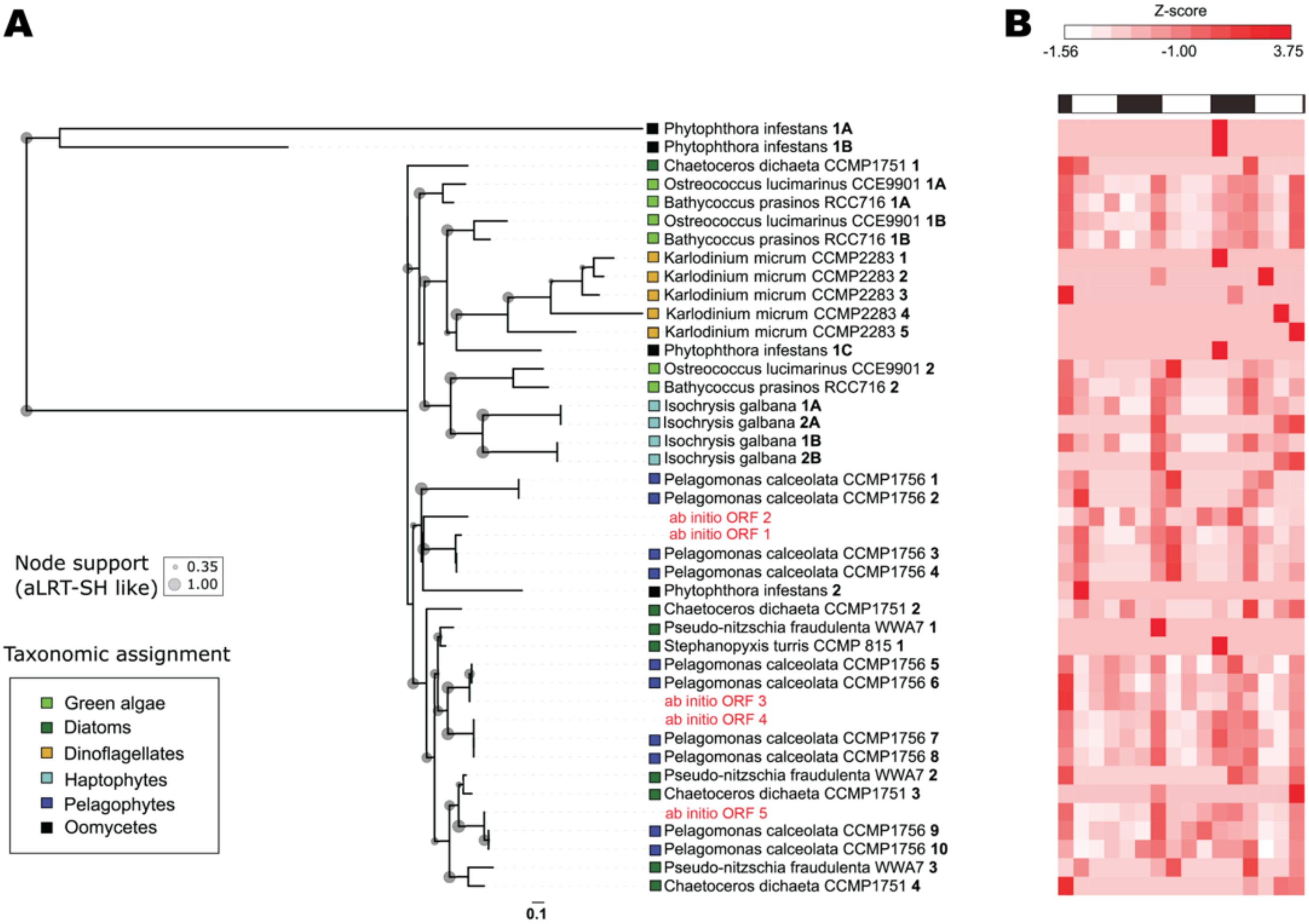
A
B
Z-score
-1.56
-1.00
3.75
Phytophthora infestans 1A
Phytophthora infestans 1B
Chaetoceros dichaeta CCMP1751 1
Ostreococcus lucimarinus CCE9901 1A
Bathycoccus prasinos RCC716 1A
Ostreococcus lucimarinus CCE9901 1B
Bathycoccus prasinos RCC716 1B
Karlodinium micrum CCMP2283 1
Karlodinium micrum CCMP2283 2
Karlodinium micrum CCMP2283 3
Karlodinium micrum CCMP2283 4
Karlodinium micrum CCMP2283 5
Phytophthora infestans 1C
Ostreococcus lucimarinus CCE9901 2
Bathycoccus prasinos RCC716 2
Isochrysis galbana 1A
Isochrysis galbana 2A
Isochrysis galbana 1B
Isochrysis galbana 2B
Pelagomonas calceolata CCMP1756 1
Pelagomonas calceolata CCMP1756 2
ab initio ORF 2
ab initio ORF 1
Pelagomonas calceolata CCMP1756 3
Pelagomonas calceolata CCMP1756 4
Phytophthora infestans 2
Chaetoceros dichaeta CCMP1751 2
Pseudo-nitzschia fraudulenta WWA7 1
Stephanopyxis turris CCMP 815 1
Pelagomonas calceolata CCMP1756 5
Pelagomonas calceolata CCMP1756 6
ab initio ORF 3
ab initio ORF 4
Pelagomonas calceolata CCMP1756 7
Pelagomonas calceolata CCMP1756 8
Pseudo-nitzschia fraudulenta WWA7 2
Chaetoceros dichaeta CCMP1751 3
ab initio ORF 5
Pelagomonas calceolata CCMP1756 9
Pelagomonas calceolata CCMP1756 10
Pseudo-nitzschia fraudulenta WWA7 3
Chaetoceros dichaeta CCMP1751 4
Node support
(aLRT-SH like)
0.35
1.00
Taxonomic assignment
Green algae
Diatoms
Dinoflagellates
Haptophytes
Pelagophytes
Oomycetes
0.1

**c**	**Transcript ID**	**Pfam domain ID**	**Domain description**	**Corresponding genus & species IDs on the LOV tree**
**Reference transcripts**	CAMPEP_0199691428-CAMNT_0045556983	PF07716, PF13426	bZIP_2 (basic leucine zipper), PAS_9	Pelagomonas calceolata CCMP1756 **6**
	CAMPEP_0199699820-CAMNT_0045565857	PF13426	PAS_9	Pelagomonas calceolata CCMP1756 **4**
	CAMPEP_0199705234-CAMNT_0045571731	PF07716, PF13426	bZIP_2 (basic leucine zipper), PAS_9	Pelagomonas calceolata CCMP1756 **10**
	CAMPEP_0199709614-CAMNT_0045576641	PF07716, PF13426	bZIP_2 (basic leucine zipper), PAS_9	Pelagomonas calceolata CCMP1756 **8**
	CAMPEP_0199710938-CAMNT_0045577979	PF13426	PAS_9	Pelagomonas calceolata CCMP1756 **2**
	contig_10685_301_1296-Pelagomonas	PF13426	PAS_9	Pelagomonas calceolata CCMP1756 **3**
	contig_12486_283_1311-Pelagomonas	PF07716, PF13426	bZIP_2 (basic leucine zipper)	Pelagomonas calceolata CCMP1756 **7**
	contig_40022_3223_3810-Pelagomonas	PF13426	PAS_9	Pelagomonas calceolata CCMP1756 **9**
	contig_5472_151_1554-Pelagomonas	PF13426	PAS_9	Pelagomonas calceolata CCMP1756 **1**
	contig_8318_666_1631-Pelagomonas	PF07716, PF13426	bZIP_2 (basic leucine zipper), PAS_9	Pelagomonas calceolata CCMP1756 **5**
	GGTG_05190T0-supercont13	PF13426, PF08447, PF00320	PAS_9, PAS_3, GATA (GATA zinc finger domain)	Phytophthora infestans **1A, 1B, 1C**
	GGTG_12596T0-supercont19	PF13426	PAS_9	Phytophthora infestans **2**
	Karlodinium-micrum-CCMP2283-20140214|17716_1	PF13426	PAS_9	Karlodinium micrum, Strain CCMP2283 **3**
	Karlodinium-micrum-CCMP2283-20140214|19826_1	PF13426, PF00069	PAS_9, Protein kinase domain	Karlodinium micrum, Strain CCMP2283 **4**
	Karlodinium-micrum-CCMP2283-20140214|23659_1	PF13426	PAS_9	Karlodinium micrum, Strain CCMP2283 **2**
	Karlodinium-micrum-CCMP2283-20140214|29782_1	PF00069, PF13426	Protein kinase domain, PAS_9	Karlodinium micrum, Strain CCMP2283 **5**
	Karlodinium-micrum-CCMP2283-20140214|4888_1	PF13426	PAS_9	Karlodinium micrum, Strain CCMP2283 **1**
	MMETSP0123-20130129|11670_1	PF13426	PAS_9	Isochrysis galbana **2A, 2B**
	MMETSP0123-20130129|18638_1	PF13426	PAS_9	Isochrysis galbana **1A, 1B**
	MMETSP0794_2-20130614|17546_1	PF07716, PF13426	bZIP_2 (basic leucine zipper), PAS_9	Stephanopyxis turris CCMP 815 **1**
	MMETSP1447-20131203|31680_1	PF00170, PF13426	Basic leucine zipper (bZIP_1), PAS_9	Chaetoceros dichaeta CCMP1751 **3**
	MMETSP1447-20131203|5598_1	PF13426	PAS_9	Chaetoceros dichaeta CCMP1751 **1**
	MMETSP1447-20131203|66049_1	PF07716, PF13426	bZIP_2 (basic leucine zipper), PAS_9	Chaetoceros dichaeta CCMP1751 **4**
	MMETSP1447-20131203|9341_1	PF07716, PF13426	bZIP_2 (basic leucine zipper), PAS_9	Chaetoceros dichaeta CCMP1751 **2**
	MMETSP1460-20131121|1537_1	PF13426, PF00069	PAS_9, Protein kinase domain	Bathycoccus prasinos RCC716 **1A, 1B**
	MMETSP1460-20131121|31452_1	PF13426, PF00512, PF02518, PF00072	PAS_9, HisKA (Histidine kinase), GHKL (Gyrase-Hsp90-Histidine Kinase-MutL), Response regulator receiver domain	Bathycoccus prasinos, Strain RCC716 **2**
	OSTLU_35077-NC_009363	PF13426	PAS_9	Ostreococcus lucimarinus CCE9901 **2**
	OSTLU_40751-NC_009369	PF13426, PF00069	PAS_9, Protein kinase domain	Ostreococcus lucimarinus CCE9901 1A &1[illegible]
	Pseudo_nitzschia-fradulenta-WWA7-20140214|1617_1	PF00170, PF13426	Basic leucine zipper (bZIP_1), PAS_9	Pseudo-nitzschia fraudulenta WWA7 **3**
	Pseudo_nitzschia-fradulenta-WWA7-20140214|45782_1	PF00170, PF13426	Basic leucine zipper (bZIP_1), PAS_9	Pseudo-nitzschia fraudulenta WWA7 **2**
	Pseudo_nitzschia-fradulenta-WWA7-20140214|86850_1	PF00170, PF13426	Basic leucine zipper (bZIP_1), PAS_9	Pseudo-nitzschia fraudulenta WWA7 **1**
***ab initio* ORFs**	contig_318056_1_312_+	PF13426	PAS_9	*ab initio* ORF 1
	contig_595760_1_551_-	PF13426	PAS_9	*ab initio* ORF 2
	contig_608828_30_709_-	PF07716, PF13426	bZIP_2 (basic leucine zipper), PAS_9	*ab initio* ORF 3
	contig_620084_102_530_+	PF13426	PAS_9	*ab initio* ORF 4
	contig_492140_1_541_-	PF13426	PAS_9	*ab initio* ORF 5

Figure S14 (A) Maximum likelihood phylogenetic tree of the LOV domains from select *ab initio* ORFs and reference transcripts. Branch labels indicate the species of origin of the reference transcripts (in black). "*ab initio"* prefix refers to transcripts assembled directly from the metatranscriptomic datasets (in red). Numeric suffixes added to the labels (in bold letters) indicate the number of LOV domain transcripts present in each species. Several transcripts harbor multiple LOV domains which are denoted by an additional suffix (A, B, C). For example, one transcript from *Phytophthora infestans* harbors three LOV domains and all of these are shown on the tree. Colored squares denote the taxonomic affiliations of the reference transcripts. **(B)** Expression profile of the reference transcripts and *ab initio* ORFs along the sampling period as Z-scores. Night and day periods are denoted by dark and light bars above the heatmap. **(C)** Table indicating the transcript ID, Pfam annotation, and corresponding taxonomic information for LOV domain containing reference and *ab initio* ORFs. Several lineages of eukaryotic phytoplankton showed ORFs possessing LOV (Light-Oxygen-Voltage) domains with peak activity just before dawn. LOV domains respond to blue light (12) and well-characterized LOV domain containing proteins are known to convert photosensory stimuli into downstream biochemical signal (13) via adjacent effector domains like serine-threonine kinases (in case of phototropins) or basic leucine zipper (b-ZIP) transcription regulatory domains (in case of Aureochromes) (14). In addition, a large number of novel LOV- effector domain combinations have been previously identified across the tree of life (15). Consistent with previous observations, we found aureochrome-like domain combinations in pelagophytes (16) and diatoms (17) and a phototropin like domain combination (LOV-protein kinase) in *Ostreococcus* (18) and *Bathycoccus*. Although presence and possible function of LOV domain containing proteins have not been discussed in dinoflagellates or oomycetes, we detected LOV-protein kinase domain combinations in *Karlodinium* and a GATA zinc finger – LOV combination in oomycetes *Phytophthora*. However, the expressions of these proteins were very low and did not follow a clear diel pattern. A vast majority of the reference and *ab initio* transcripts had peak expression at dawn, irrespective of the domain combinations, indicating a common light-regulated signaling/transcriptional response mediated by the LOV domain in these organisms.

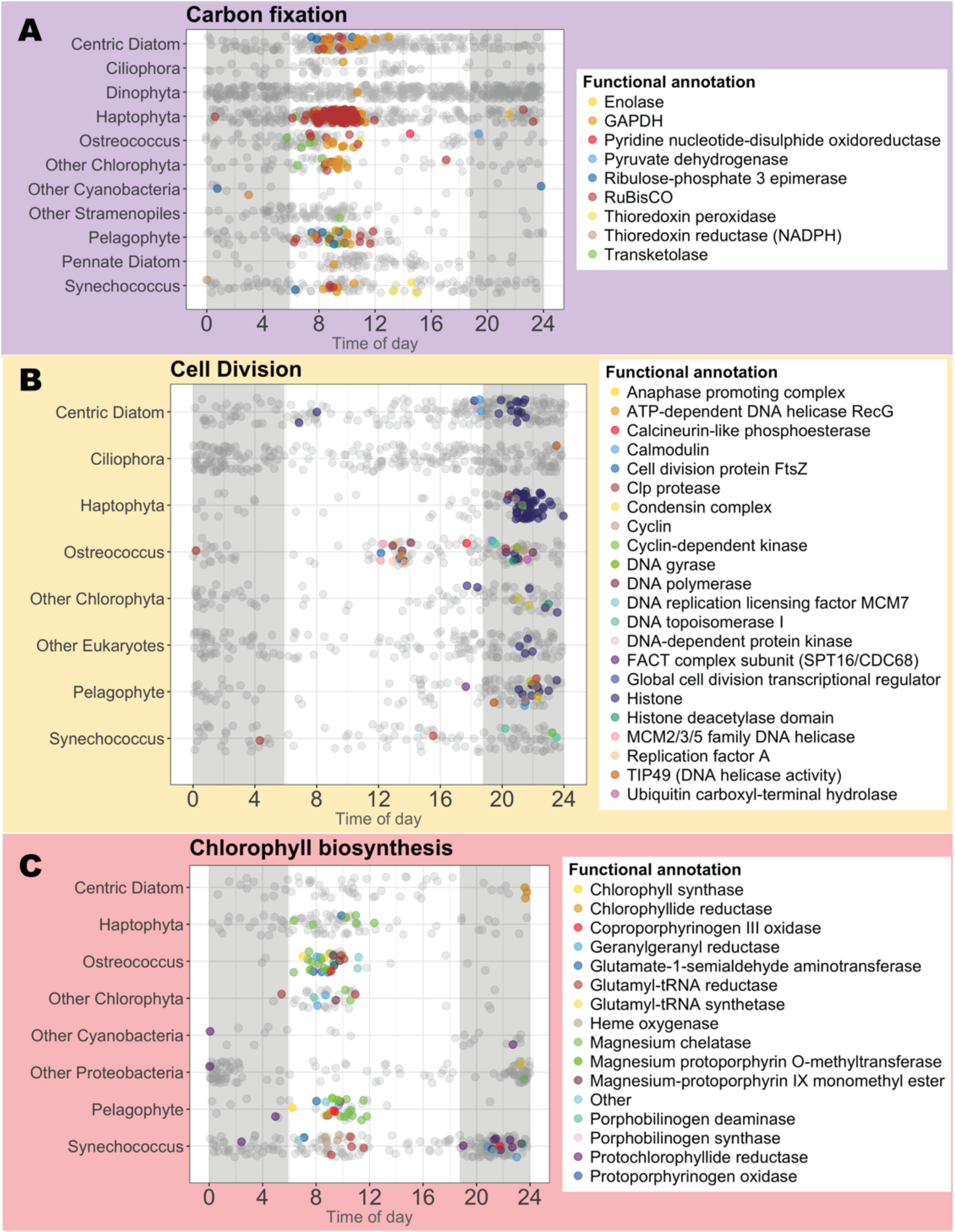
A
Carbon fixation
Centric Diatom
Ciliophora
Dinophyta
Haptophyta
Ostreococcus
Other Chlorophyta
Other Cyanobacteria
Other Stramenopiles
Pelagophyte
Pennate Diatom
Synechococcus
0
4
8
12
16
20
24
Time of day
Functional annotation
Enolase
GAPDH
Pyridine nucleotide-disulphide oxidoreductase
Pyruvate dehydrogenase
Ribulose-phosphate 3 epimerase
RuBisCO
Thioredoxin peroxidase
Thioredoxin reductase (NADPH)
Transketolase
B
Cell Division
Centric Diatom
Ciliophora
Haptophyta
Ostreococcus
Other Chlorophyta
Other Eukaryotes
Pelagophyte
Synechococcus
0
4
8
12
16
20
24
Time of day
Functional annotation
Anaphase promoting complex
ATP-dependent DNA helicase RecG
Calcineurin-like phosphoesterase
Calmodulin
Cell division protein FtsZ
Clp protease
Condensin complex
Cyclin
Cyclin-dependent kinase
DNA gyrase
DNA polymerase
DNA replication licensing factor MCM7
DNA topoisomerase I
DNA-dependent protein kinase
FACT complex subunit (SPT16/CDC68)
Global cell division transcriptional regulator
Histone
Histone deacetylase domain
MCM2/3/5 family DNA helicase
Replication factor A
TIP49 (DNA helicase activity)
Ubiquitin carboxyl-terminal hydrolase
C
Chlorophyll biosynthesis
Centric Diatom
Haptophyta
Ostreococcus
Other Chlorophyta
Other Cyanobacteria
Other Proteobacteria
Pelagophyte
Synechococcus
0
4
8
12
16
20
24
Time of day
Functional annotation
Chlorophyll synthase
Chlorophyllide reductase
Coproporphyrinogen III oxidase
Geranylgeranyl reductase
Glutamate-1-semialdehyde aminotransferase
Glutamyl-tRNA reductase
Glutamyl-tRNA synthetase
Heme oxygenase
Magnesium chelatase
Magnesium protoporphyrin O-methyltransferase
Magnesium-protoporphyrin IX monomethyl ester
Other
Porphobilinogen deaminase
Porphobilinogen synthase
Protochlorophyllide reductase
Protoporphyrinogen oxidase

Figure S15 Peak expression time of large fraction ORFs involved in **(A)** carbon fixation, **(B)** cell division, and **(C)** chlorophyll biosynthesis. Night is indicated by grey shading, while white represents daylight hours. Significantly periodic ORFs (HRA; FDR adjusted $p \leq 0.1$) are colored by functional annotation; insignificant ORFs are shown in grey.

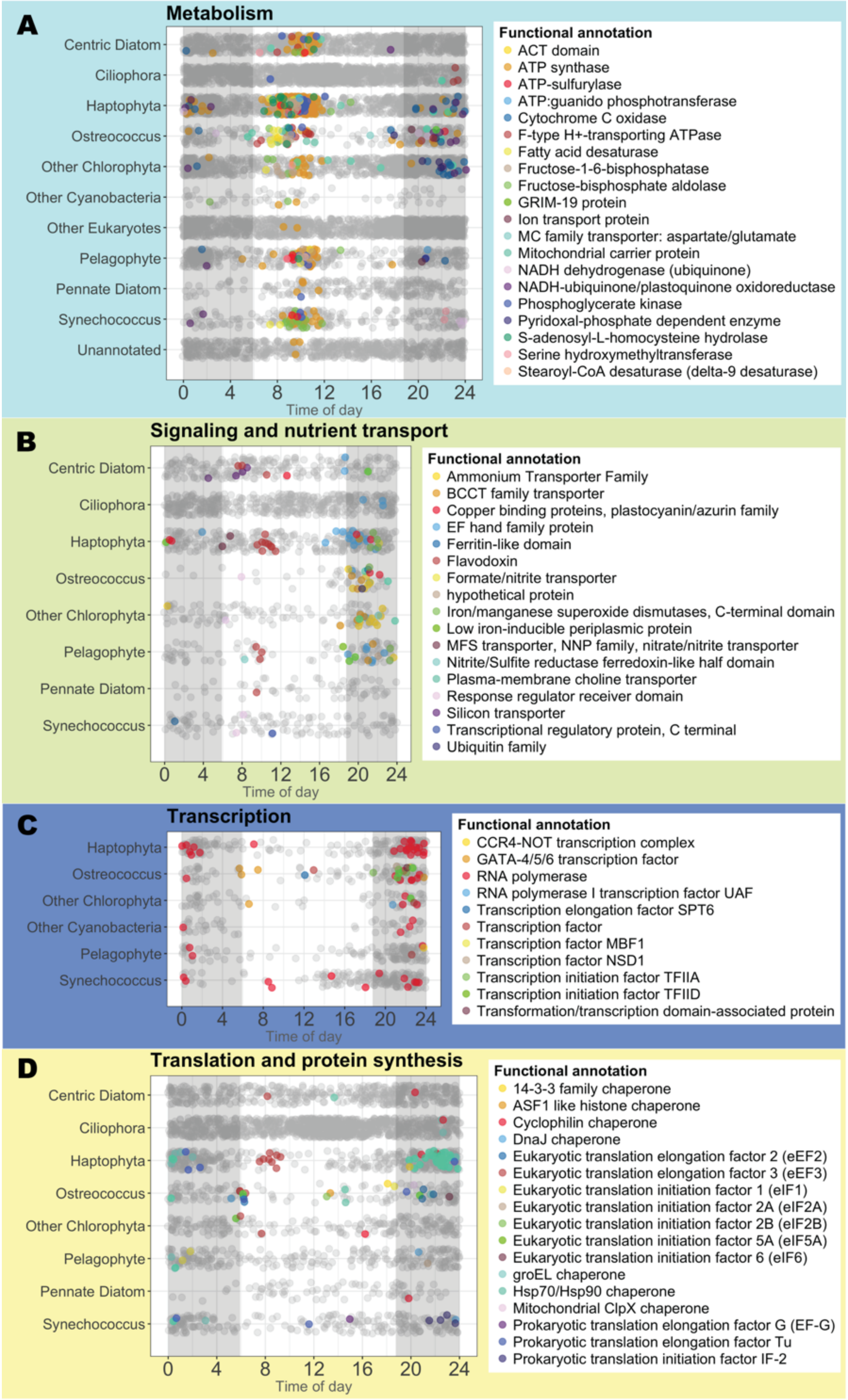
A
Metabolism
Centric Diatom
Ciliophora
Haptophyta
Ostreococcus
Other Chlorophyta
Other Cyanobacteria
Other Eukaryotes
Pelagophyte
Pennate Diatom
Synechococcus
Unannotated
0 4 8 12 16 20 24
Time of day
Functional annotation
ACT domain
ATP synthase
ATP-sulfurylase
ATP:guanido phosphotransferase
Cytochrome C oxidase
F-type H+-transporting ATPase
Fatty acid desaturase
Fructose-1-6-bisphosphatase
Fructose-bisphosphate aldolase
GRIM-19 protein
Ion transport protein
MC family transporter: aspartate/glutamate
Mitochondrial carrier protein
NADH dehydrogenase (ubiquinone)
NADH-ubiquinone/plastoquinone oxidoreductase
Phosphoglycerate kinase
Pyridoxal-phosphate dependent enzyme
S-adenosyl-L-homocysteine hydrolase
Serine hydroxymethyltransferase
Stearoyl-CoA desaturase (delta-9 desaturase)
B
Signaling and nutrient transport
Centric Diatom
Ciliophora
Haptophyta
Ostreococcus
Other Chlorophyta
Pelagophyte
Pennate Diatom
Synechococcus
0 4 8 12 16 20 24
Time of day
Functional annotation
Ammonium Transporter Family
BCCT family transporter
Copper binding proteins, plastocyanin/azurin family
EF hand family protein
Ferritin-like domain
Flavodoxin
Formate/nitrite transporter
hypothetical protein
Iron/manganese superoxide dismutases, C-terminal domain
Low iron-inducible periplasmic protein
MFS transporter, NNP family, nitrate/nitrite transporter
Nitrite/Sulfite reductase ferredoxin-like half domain
Plasma-membrane choline transporter
Response regulator receiver domain
Silicon transporter
Transcriptional regulatory protein, C terminal
Ubiquitin family
C
Transcription
Haptophyta
Ostreococcus
Other Chlorophyta
Other Cyanobacteria
Pelagophyte
Synechococcus
0 4 8 12 16 20 24
Time of day
Functional annotation
CCR4-NOT transcription complex
GATA-4/5/6 transcription factor
RNA polymerase
RNA polymerase I transcription factor UAF
Transcription elongation factor SPT6
Transcription factor
Transcription factor MBF1
Transcription factor NSD1
Transcription initiation factor TFIIA
Transcription initiation factor TFIID
Transformation/transcription domain-associated protein
D
Translation and protein synthesis
Centric Diatom
Ciliophora
Haptophyta
Ostreococcus
Other Chlorophyta
Pelagophyte
Pennate Diatom
Synechococcus
0 4 8 12 16 20 24
Time of day
Functional annotation
14-3-3 family chaperone
ASF1 like histone chaperone
Cyclophilin chaperone
DnaJ chaperone
Eukaryotic translation elongation factor 2 (eEF2)
Eukaryotic translation elongation factor 3 (eEF3)
Eukaryotic translation initiation factor 1 (eIF1)
Eukaryotic translation initiation factor 2A (eIF2A)
Eukaryotic translation initiation factor 2B (eIF2B)
Eukaryotic translation initiation factor 5A (eIF5A)
Eukaryotic translation initiation factor 6 (eIF6)
groEL chaperone
Hsp70/Hsp90 chaperone
Mitochondrial ClpX chaperone
Prokaryotic translation elongation factor G (EF-G)
Prokaryotic translation elongation factor Tu
Prokaryotic translation initiation factor IF-2

Figure S16 Peak expression time of large fraction ORFs involved in **(A)** metabolism, **(B)** signaling and nutrient transport, **(C)** transcription and **(D)** translation and protein synthesis. Night is indicated by grey shading. Significantly periodic ORFs (HRA; FDR adjusted $p \leq 0.1$) are colored by functional annotation; insignificant ORFs are shown in grey. Several eukaryotic translation elongation factor 3 (eEF3) *ab initio* ORFs detected in the large size class were significantly periodic (dark red). eEF3 presents a novel peptide synthesis mechanism for phytoplankton. eEF3 was previously thought to be unique to fungi, but homologs have been recently discovered in various phytoplankton lineages, and one haptophyte (*Phytopthora infestans*) eEF3 was proven capable of restoring function in yeast (19). Of the 122 eEF3 ORFs in our data, the majority belonged to dinoflagellates, but several were also found among haptophytes (9 ORFs), chlorophytes (9), centric (7) and pennate (3) diatoms, pelagophytes (4), other stramenopiles (1) and even ciliates (1).

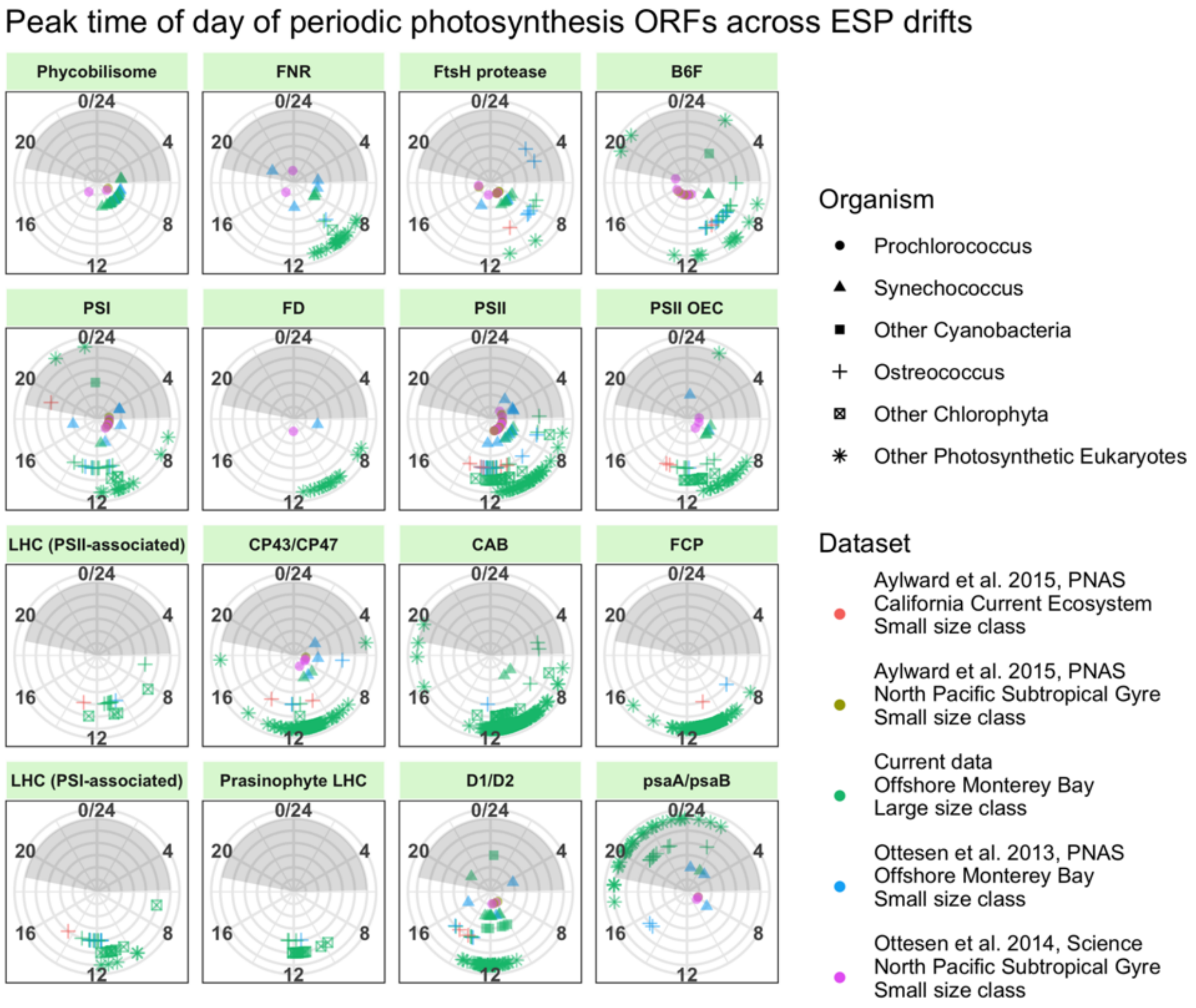

Figure S17 Comparison of peak expression time of photosynthesis related ORFs between the current data and previously studied ESP drift tracks (1,20,21). Taxa groups are distinguished by shape (legend, top right) and radius (innermost: *Prochlorococcus*, outermost: “Other Photosynthetic Eukaryotes”). Colors indicate dataset of origin. Night (as observed for current data) is indicated by grey shading. In some cases, addition of picoplankton data from other environments revealed a difference in timing between prokaryotic and eukaryotic photosynthetic proteins. For example, cyanobacterial PSII, CP43/CP47, and PSII OEC peak earlier than their equivalents in photosynthetic eukaryotes, with *Ostreococcus* and other chlorophytes peaking last, and cyanobacterial FtsH and B6F peak earlier than equivalents in photosynthetic eukaryotes.

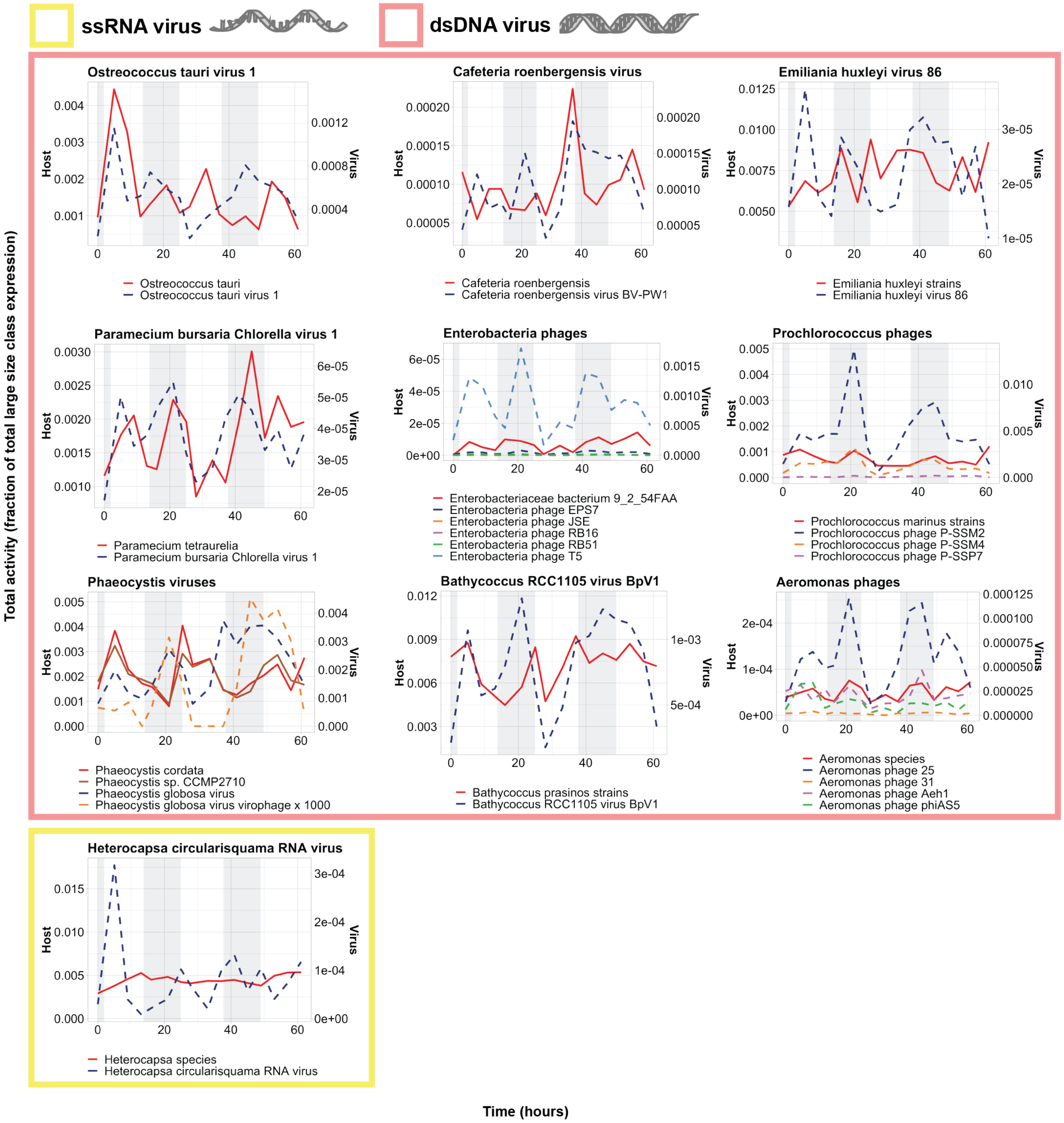

Figure S18 Continuation of Figure 6: virus/host dynamics in the large size class. Viruses and hosts are annotated as the closest reference available in our database, as determined by LPI. Library normalized expression of ORFs classified as ssRNA (yellow) and dsDNA (pink) viruses and their putative hosts by LPI are shown. Putative host expression is represented by solid lines and corresponds to left y-axes; virus expression is represented by dashed lines and corresponds to the right y-axes. *Phaeocystis globulosa* virus virophage expression was multiplied by 10^3 for better visualization. Night hours are shaded in grey.

References

1. Ottesen EA, Young CR, Eppley JM, Ryan JP, Chavez FP, Scholin CA, et al. Pattern and synchrony of gene expression among sympatric marine microbial populations. Proc Natl Acad Sci. 2013 Feb 5;110(6):E488–97.
2. Li H, Durbin R. Fast and accurate short read alignment with Burrows-Wheeler transform. Bioinformatics. 2009 Jul 15;25(14):1754–60.
3. Bruland KW, Rue EL, Smith GJ. Iron and macronutrients in California coastal upwelling regimes: Implications for diatom blooms. Limnol Oceanogr. 2001 Nov;46(7):1661–74.
4. Carradec Q, Pelletier E, Da Silva C, Alberti A, Seeleuthner Y, Blanc-Mathieu R, et al. A global ocean atlas of eukaryotic genes. Nat Commun. 2018;9(1).
5. Hutchins DA, DiTullio GR, Zhang Y, Bruland KW. An iron limitation mosaic in the California upwelling regime. Limnol Oceanogr. 1998;43(6):1037–54.
6. Johnson KS, Chavez FP, Elrod VA, Fitzwater SE, Pennington JT, Buck KR, et al. The annual cycle of iron and the biological response in Central California coastal waters. Geophys Res Lett. 2001;28(7):1247–50.
7. Simmons MP, Sudek S, Monier A, Limardo AJ, Jimenez V, Perle CR, et al. Abundance and biogeography of picoprasinophyte ecotypes and other phytoplankton in the eastern North Pacific Ocean. Appl Environ Microbiol. 2016;
8. Siano R, Montresor M, Probert I, Not F, de Vargas C. Pelagodinium gen. nov. and P. béii comb. nov., a dinoflagellate symbiont of planktonic foraminifera. Protist. 2010;161(3):385–99.
9. Töpel M, Pinder MIM, Johansson ON, Kourtchenko O, Godhe A, Clarke AK. Complete genome sequence of Loktanella vestfoldensis strain SMR4r, a novel strain isolated from a culture of the chain- forming diatom Skeletonema marinoi. Genome Announc. 2018;6(12):e01558-17.
10. Bloh AH, Usup G, Ahmad A. Loktanella spp. Gb03 as an algicidal bacterium, isolated from the culture of Dinoflagellate Gambierdiscus belizeanus. Vet World. 2016;9(2):142–6.
11. Pelve EA, Fontanez KM, DeLong EF. Bacterial succession on sinking particles in the ocean's interior. Front Microbiol. 2017;8(NOV):2269.
12. Christie JM, Salomon M, Nozue K, Wada M, Briggs WR. LOV (light, oxygen, or voltage) domains of the blue-light photoreceptor phototropin (nph1): Binding sites for the chromophore flavin mononucleotide. Proc Natl Acad Sci. 1999;96(15):8779–83.
13. Christie JM. Phototropin Blue-Light Receptors. Annu Rev Plant Biol. 2007;58(1):21–45.
14. Takahashi F, Yamagata D, Ishikawa M, Fukamatsu Y, Ogura Y, Kasahara M, et al. Aureochrome, a photoreceptor required for photomorphogenesis in stramenopiles. Proc Natl Acad Sci. 2007;104(49):19625–30.
15. Glantz ST, Carpenter EJ, Melkonian M, Gardner KH, Boyden ES, Wong GK-S, et al. Functional and topological diversity of LOV domain photoreceptors. Proc Natl Acad Sci. 2016;113(11):E1442–51.
16. Ishikawa M, Takahashi F, Nozaki H, Nagasato C, Motomura T, Kataoka H. Distribution and phylogeny of the blue light receptors aureochromes in eukaryotes. Planta. 2009;230(3):543–52.
17. Schellenberger Costa B, Sachse M, Jungandreas A, Bartulos CR, Gruber A, Jakob T, et al. Aureochrome 1a Is Involved in the Photoacclimation of the Diatom Phaeodactylum tricornutum. PLoS One. 2013;8(9).
18. Kianianmomeni A, Hallmann A. Algal photoreceptors: In vivo functions and potential applications. Vol. 239, Planta. 2014. p. 1–26.
19. Mateyak MK, Pupek JK, Garino AE, Knapp MC, Colmer SF, Kinzy TG, et al. Demonstration of translation elongation factor 3 activity from a non-fungal species, Phytophthora infestans. PLoS One. 2018;13(1):1–14.
20. Aylward FO, Eppley JM, Smith JM, Chavez FP, Scholin CA, DeLong EF. Microbial community transcriptional networks are conserved in three domains at ocean basin scales. Proc Natl Acad Sci. 2015;112(17):5443–8.

21. Ottesen EA, Young CR, Gifford SM, Eppley JM, Marin R, Schuster SC, et al. Multispecies diel transcriptional oscillations in open ocean heterotrophic bacterial assemblages. Science (80-). 2014;345(6193).
