# Supplementary material for: Diel transcriptional response of a California Current plankton microbiome to light, low iron, and enduring viral infection
Source: ISME J. 2019 Jul 18;13(11):2817–33. doi: 10.1038/s41396-019-0472-2 (PMC6794264; doi:10.1038/s41396-019-0472-2)
Supplement: Supplementary file 3 — S3: Description of supplemental datasets [file 41396_2019_472_MOESM3_ESM.pdf]

Supplementary File 3

Description of Supplementary Datasets

Supplementary Data 1: Summary statistics for assembly and taxonomic annotation of large **(sheet A)** and small **(sheet B)** size class metatranscriptomes

Supplementary Data 2: Summary statistics for large fraction rRNA amplicon analysis pipeline by library **(sheet A)** and overall **(sheet B)**

Supplementary Data 3: 18S rRNA amplicon data for the large size class
Sheet A: Raw count data for each unique 18S sequence
Sheet B: Library (time point) normalized count data for each unique 18S sequence
Sheet C-F: Library (time point) normalized count data summarized by species, family, order, and phylum, respectively

Supplementary Data 4: Non-plastid 16S rRNA amplicon data for the large size class
Sheet A: Raw count data for each unique 16S sequence
Sheet B: Library (time point) normalized count data for each unique 16S sequence
Sheet C-F: Library (time point) normalized count data summarized by species, family, order, and phylum, respectively

Supplementary Data 5: 16S rRNA plastid amplicon data for the large size class
Sheet A: Raw count data for each unique 16S sequence
Sheet B: Library (time point) normalized count data for each unique 16S sequence
Sheet C-F: Library (time point) normalized count data summarized by species, family, order, and phylum, respectively

Supplementary Data 6: Raw count data for large fraction *ab initio* nuclear and organellar ORFs with taxonomic annotations, functional annotations, and HRA results. (Note- large file, best viewed in RStudio or similar rather than excel)

Supplementary Data 7: Raw count data resulting from nucleotide space mapping of large and small size class metatranscriptome reads to chosen references. (Note- large file, best viewed in RStudio or similar rather than excel)

Supplementary Data 8: Raw count data for clusters of *ab initio* and reference ORFs (as determined by hierarchical clustering of amino acid sequences). Cluster statistics and consensus annotations are included. Clusters differentially expressed between large and small fraction (EdgeR FDR $p < 0.05$) are also noted. (Note- large file, best viewed in RStudio or similar rather than excel)

Supplementary Data 9: Differential expression of taxa groups between large and small fraction (EdgeR FDR $p < 0.05$) using *ab initio* ORFs.
Sheet A: Using the 15 taxa groups shown in main text figures for ease of viewing
Sheet B: Using 26 taxa groups for a higher resolution picture

Supplementary Data 10: Differential expression of genera between large and small fraction (EdgeR FDR $p < 0.05$) using *ab initio* ORFs.

Supplementary Data 11: Descriptions of column names for previous supplementary data files

Supplementary Data 12: Environmental metadata
Sheet A: ESP drift track coordinates and velocity
Sheet B: Bottle data (chlorophyll and nutrients) as measured from shipboard CTD/niskin rosette deployments
Sheet C: Pressure, temperature, salinity, corrected chlorophyll, and percent transmission as measured from shipboard CTD/niskin rosette deployments
Sheet D: Surface photosynthetically active radiation (PAR) as measured from the surface drifter
Sheet E: Previous iron measurements in the region of the ESP drift
